# Immunosuppressive cells as barriers to cancer therapy: mechanisms and emerging solutions

**DOI:** 10.3389/fimmu.2026.1896156

**Published:** 2026-08-24

**Authors:** Mengmeng Liu, Yichen Zhu, Hemiao Liang, Yu Li, Huaimin Liu, Jia Li

**Affiliations:** 1 Department of Integrated Chinese and Western Medicine, Affiliated Cancer Hospital of Zhengzhou University and Henan Cancer Hospital, Zhengzhou, China; 2 Department of Internal Medicine, Affiliated Cancer Hospital of Zhengzhou University and Henan Cancer Hospital, Zhengzhou, China; 3 Department of Neurology, The Eighth Medical Center, Chinese People’s Liberation Army (PLA) General Hospital, Beijing, China

**Keywords:** combination strategies, immunosuppressive cells, immunotherapy resistance, metabolic reprogramming, tumor microenvironment

## Abstract

Tumor microenvironment-resident immunosuppressive cells-comprising myeloid-derived suppressor cells, regulatory T cells, and tumor-associated macrophages-constitute primary obstacles to effective cancer immunotherapy. Advances in single-cell and spatial multi-omics have uncovered their substantial functional heterogeneity, tissue-adaptive reprogramming, and organ-specific architectures distinguishing primary tumors from metastatic lesions. Beyond canonical immune checkpoint pathways, non-canonical regulatory layers--including metabolic-immune crosstalk, epigenetic regulation, microbiome-mediated distant signaling, and therapy-induced adaptive remodeling-further reinforce treatment resistance. Based on these mechanistic insights, systematic synergistic strategies have been developed, such as multi-pathway checkpoint blockade, ADC-immunotherapy combinations, temporally and spatially optimized conventional therapies, and targeted agents that deplete or reprogram suppressive populations. Emerging biomarkers, repurposed pharmaceuticals, and pan-cancer therapeutic principles are refining patient stratification and combination regimens. This review offers a comprehensive framework for understanding and surmounting immunosuppressive barriers in cancer therapy.

## 1 Introduction

Cancer immunotherapy has revolutionized the treatment landscape of malignant tumors, with immune checkpoint inhibitors (ICIs) and chimeric antigen receptor (CAR) T-cell therapies achieving unprecedented clinical success. ICIs targeting the PD-1/PD-L1 and CTLA-4 pathways have transformed the prognosis of multiple malignancies, increasing the 5-year survival rate of metastatic melanoma from less than 5% to approximately 50% and establishing long-term survival benefits in non-small cell lung cancer, renal cell carcinoma, and other solid tumors (1, 2). However, despite these remarkable advances, primary and acquired resistance remains a major clinical challenge, with objective response rates remaining below 20% in many cancer types (2). Similarly, while CAR-T cell therapies have achieved remarkable curative effects in hematological malignancies, their efficacy in solid tumors is severely limited by multiple barriers within the tumor microenvironment (TME) (3).

A growing body of evidence indicates that immunosuppressive cells in the TME, including myeloid-derived suppressor cells (MDSCs), regulatory T cells (Tregs), and tumor-associated macrophages (TAMs), are the primary drivers of immunotherapy resistance. Traditionally, these cells were viewed as homogeneous populations with uniform immunosuppressive functions. However, the rapid development of single-cell sequencing and spatial omics technologies has revolutionized our understanding of these cells, revealing their extraordinary functional heterogeneity and tissue-specific characteristics (4, 5). Recent studies have identified multiple functionally distinct subsets of immunosuppressive cells with unique molecular signatures, spatial distribution patterns, and biological effects, which differentially contribute to tumor progression and therapeutic response (6). Furthermore, the immunosuppressive TME is not a static entity but a dynamic network shaped by complex intercellular crosstalk between immune cells, stromal cells, and tumor cells, as well as systemic factors such as the gut microbiome (7).

Beyond canonical signaling pathways, emerging research has uncovered multiple non-canonical regulatory mechanisms that modulate immunosuppressive cell function and drive therapy resistance. Metabolic reprogramming in the TME creates a nutrient-deprived and acidic environment that preferentially supports the survival and function of immunosuppressive cells while inhibiting effector T cells (8, 9). Epigenetic modifications and non-coding RNAs play critical roles in regulating the differentiation and plasticity of immunosuppressive cells. Notably, therapy-induced adaptive remodeling of immunosuppressive cells has emerged as a key mechanism of acquired resistance, where ICIs, chemotherapy, and radiotherapy can paradoxically induce the expansion and activation of immunosuppressive cell subsets, limiting the durability of therapeutic responses (10, 11).

The deepening understanding of immunosuppressive cell heterogeneity and regulatory mechanisms has paved the way for the rational design of systematic synergistic intervention strategies. Multi-pathway immune checkpoint blockade, combination of immunotherapy with antibody-drug conjugates (ADCs), temporal-spatial optimization of conventional therapies, cross-dimensional modulation of the metabolism-immunity-microbiota axis, and synergistic enhancement of cell therapies have all shown promising clinical results (12). Moreover, the development of precision biomarkers has enabled the stratification of patients into distinct subgroups, guiding individualized treatment decisions and improving therapeutic outcomes (13, 14).

This review advances a network-based framework for immunosuppressive cell biology in cancer therapy resistance. Rather than treating MDSCs, Tregs, TAMs and additional suppressive populations as isolated entities, we organize their effects around convergent resistance modules: metabolic competition, checkpoint-mediated inhibition, impaired antigen presentation, stromal/physical exclusion and therapy-induced remodeling. We then examine how these modules are conditioned by tissue context and metastatic adaptation, and how non-canonical regulatory layers—including metabolic–immune crosstalk, epigenetic regulation, microbiome-mediated signaling and treatment-induced adaptation—can be translated into mechanism-matched intervention strategies. Finally, we distinguish established clinical observations from emerging preclinical concepts and discuss biomarker-guided implementation and translational constraints.

## 2 Heterogeneity and plasticity of immunosuppressive cells: from uniform populations to functional subsets and tissue adaptation

### 2.1 Functional subsets of core immunosuppressive cells

Interpretive framework and limitations of cell-state assignment. Functional labels such as MDSC, TAM and Treg are useful organizing concepts, but they should not be equated with fixed, universally conserved cell identities. Marker combinations, tissue processing, species, treatment exposure and spatial context can alter the apparent abundance and state of these populations. In particular, single-cell or spatial transcriptomic signatures alone support a cell-state hypothesis rather than definitive suppressive function; functional assays, orthogonal protein-level validation and clinical outcome correlations are required before assigning a causal role. Throughout this review, we therefore distinguish well-supported mechanisms from context-dependent observations and emerging hypotheses.

#### 2.1.1 Myeloid-derived suppressor cells

MDSCs are a heterogeneous population arising from the aberrant differentiation of myeloid cells under pathological conditions. They represent core immunosuppressive and tumor-promoting components of the TME and play multi-dimensional, critical roles throughout the entire disease course, including tumor initiation, progression, metastasis, and therapy resistance (15–17). Classical studies have established that the core function of MDSCs is potent antitumor immunosuppression. Through high expression of arginase 1 (ARG1) and inducible nitric oxide synthase (iNOS), MDSCs produce reactive oxygen species (ROS) and reactive nitrogen intermediates (RNI), deplete amino acids essential for T cell activation, and directly suppress the antitumor activity of effector T cells and natural killer (NK) cells (18, 19). Simultaneously, MDSCs upregulate immune checkpoint molecules such as PD-L1 and secrete anti-inflammatory cytokines, thereby inducing the differentiation and expansion of regulatory Tregs and further amplifying the immunosuppressive effect within the TME (20, 21).

In recent years, advanced technologies including single-cell spatiotemporal transcriptomics and multi-omics have further elucidated the functional heterogeneity and novel regulatory mechanisms of MDSCs beyond previous knowledge. Studies using human tumor samples have identified a terminally differentiated polymorphonuclear MDSC subset characterized by CD66b^+^LOX-1^+^, which drives the formation of an immune-desert TME through collagen remodeling and serves as a core mediator of primary resistance to immune checkpoint inhibitors (22). In the realms of metabolism and epigenetic regulation, recent studies have demonstrated that glutamine metabolic reprogramming and m^6^A epigenetic modification significantly enhance the immunosuppressive function of MDSCs by modulating the STAT3 signaling axis, thereby refining the molecular network that governs MDSC function (23, 24). Furthermore, recent evidence has established that MDSCs mediate CAR-T cell exhaustion and radiotherapy resistance through the secretion of exosomal non-coding RNAs, expanding their mechanisms of action in therapy resistance (24, 25). In addition, earlier studies have confirmed that MDSCs can establish pre-metastatic niches at early tumor stages and drive tumor invasion and angiogenesis, positioning them as core markers of poor prognosis and key targets for combination immunotherapy (26, 27).

#### 2.1.2 Tumor-associated macrophages

TAMs are one of the most abundant immune cell populations in the TME, with high heterogeneity and functional plasticity. Their infiltration level is strongly associated with poor prognosis, immune escape, and therapeutic resistance in most solid tumors (28). The traditional M1/M2 bipolar classification cannot fully recapitulate the *in vivo* phenotypes of TAMs. Single-cell sequencing and spatial transcriptomic studies have divided TAMs into functional subsets including immunoregulatory, pro-angiogenic, lipid-associated, and tissue-resident subtypes (29, 30). The functions of TAMs are co-determined by developmental origin, tissue microenvironment, and metabolic reprogramming, with core functions focusing on five aspects: immunosuppression, tumor progression promotion, angiogenesis, invasion and metastasis, and therapeutic resistance (31, 32).

The core function of TAMs is to establish an immunosuppressive TME and mediate tumor immune evasion. TAMs highly express inhibitory molecules such as PD-L1, CD206, and CD163, which directly inhibit the proliferation and cytotoxic activity of CD8^+^ T cells. Meanwhile, TAMs secrete anti-inflammatory cytokines including IL-10 and TGF-β to induce the infiltration and activation of Tregs, thus amplifying the immunosuppressive network (33–35). TAMs also deplete arginine and tryptophan in the TME via upregulating arginase 1 (ARG1) and indoleamine 2,3-dioxygenase 1 (IDO1), leading to metabolic deprivation and functional exhaustion of effector T cells (36). Another studies have confirmed that TREM2+ and MARCO+ TAM subsets can directly inhibit the activation of NK cells, which are key mediators of primary resistance to immune checkpoint inhibitors (37, 38).

TAMs promote tumor invasion and distant metastasis through multiple mechanisms. TAMs secrete matrix metalloproteinases (MMPs) such as MMP2 and MMP9 to degrade the extracellular matrix, and induce epithelial-mesenchymal transition (EMT) of tumor cells to enhance their migration and invasion capacity (39, 40). In target organs such as the lung and liver, TAMs participate in the construction of pre-metastatic niches, and remodel the local microenvironment via S100A8/A9 and CXCL12 to provide favorable conditions for the colonization of circulating tumor cells (41, 42). Pro-angiogenesis is another core function of TAMs. TAMs in hypoxic regions upregulate VEGF and Ang-2 via the HIF-1α pathway to drive neovascularization, which provides channels for tumor growth and metastasis (43).

Metabolic reprogramming is a key novel mechanism regulating TAM functions. TAMs mainly rely on glycolysis and fatty acid oxidation for energy supply, and highly express scavenger receptors such as CD36 and MARCO to uptake lipids, forming a lipid droplet-enriched tumor-promoting phenotype (44, 45). Tumor-derived lactic acid further strengthens the immunosuppressive program of TAMs via HIF-1α, while glutamine metabolism rewiring maintains the stability of the tumor-promoting transcriptional program of TAMs, enabling them to function continuously in the nutrient-deficient TME (46, 47).

In addition, TAMs are widely involved in mediating resistance to chemotherapy, radiotherapy, and immunotherapy. TAMs secrete IL-6 and TGF-β to activate the anti-apoptotic pathway of tumor cells and reduce drug sensitivity, and inhibit chimeric antigen receptor T (CAR-T) cell function via metabolites and exosome signals (32, 48).

#### 2.1.3 Regulatory T cells

Tregs defined by the lineage-specifying transcription factor forkhead box P3 (Foxp3), are the central orchestrators of immunosuppression within the TME (49, 50). Single-cell and spatial multi-omics studies have consistently demonstrated that tumor-infiltrating Tregs (TI-Tregs) undergo tissue-adaptive reprogramming to acquire a highly activated, functionally specialized phenotype, driving tumor immune escape and primary/acquired resistance to immunotherapy via multi-layered, non-redundant mechanisms (51–53).

TI-Tregs exert direct immunosuppressive effects through soluble inhibitory mediators and cytokine deprivation. They secrete abundant inhibitory cytokines including IL-10, TGF-β, and IL-35, which directly suppress the proliferation and cytotoxic activity of CD8^+^ cytotoxic T lymphocytes and CD4^+^ effector T cells (51, 54). TGF-β activation is critically dependent on the surface receptor GARP, which presents latent TGF-β1 and mediates its conversion to the active form, enabling contact-dependent suppression of effector cells; GARP has been validated as a therapeutically tractable marker upregulated following radiotherapy (55). TI-Tregs also constitutively express high-affinity CD25, enabling them to sequester IL-2 from the local microenvironment and deprive effector T cells of this essential growth factor--a process governed by the CXCL12/CXCR4 axis that spatially positions Tregs to outcompete CD8^+^ T cells for IL-2 within tumor nests (56). Furthermore, the CD39/CD73 ectonucleotidase axis converts extracellular ATP into immunosuppressive adenosine, directly paralyzing effector T-cell function through adenosine A2A receptor signaling (57).

TI-Tregs amplify immunosuppression through immune checkpoint engagement, metabolic competition, and epigenetic stabilization. They highly express CTLA-4 and upregulate TIGIT, LAG-3, and TIM-3, driving T-cell exhaustion and contributing to immunotherapy resistance (58, 59). Among these, CTLA-4 and TIGIT are particularly enriched on tumor-infiltrating Tregs relative to their peripheral counterparts, as demonstrated by single-cell sequencing data, making their co-expression a distinguishing feature of intratumoral Tregs that can be exploited for selective depletion strategies (58). Metabolically, TI-Tregs sustain their survival in the nutrient-deprived TME by rewiring the LKB1–mevalonate pathway, while simultaneously depleting lipids and amino acids required for effector T-cell activation (60). Epigenetic maintenance of FOXP3 expression by EZH2 stabilizes the Treg suppressive phenotype and prevents their reprogramming into pro-inflammatory effector cells, while concurrently promoting the recruitment of TAMs and MDSCs, thereby establishing a feedforward immunosuppressive cascade that consolidates an immune-excluded “cold” TME (61).

Recent work further refines this metabolic plasticity. Lactate can reinforce Treg suppressive fitness through MOESIN lactylation and enhanced TGF-β signaling, and can support mitochondrial oxidative phosphorylation through an XBP1s–MGAT1–N-glycosylation program. These studies provide mechanistic support for lactate as more than a by-product of tumor glycolysis, while their therapeutic implications remain context-dependent (62, 63).

This literature also identifies tumor-infiltrating Treg metabolism as a therapeutic opportunity, but selectivity is essential because systemic Treg disruption can compromise immune homeostasis (64).

#### 2.1.4 Atypical suppressive populations expanding the immunosuppressive repertoire

##### 2.1.4.1 Subpopulation of immunosuppressive tumor-associated neutrophils

TANs are now recognized as functionally plastic regulators of the tumor microenvironment, exhibiting a spectrum of polarization states rather than a simple N1/N2 dichotomy. In most established tumors, TANs are skewed toward an immunosuppressive phenotype that promotes immune evasion and therapy resistance. They suppress CD8^+^ T cells and NK cells through arginase-1-mediated arginine depletion, reactive oxygen species production, and PD-L1 expression, while actively recruiting Tregs via CCL17 secretion, thereby establishing a feedforward immunosuppressive loop (65–67). Clinically, elevated TAN infiltration and a high neutrophil-to-lymphocyte ratio correlate with poor survival and reduced immune checkpoint inhibitor responsiveness across solid tumors; in gastric cancer, a TAN-low radiomic signature predicted a 1.9-fold higher disease control rate with anti-PD-1 therapy (83.9% vs. 44.1%) and significantly prolonged progression-free survival (HR = 0.162) (68).

Neutrophil extracellular traps (NETs) further amplify immune exclusion by forming physical barriers that entrap CD8^+^ T cells and suppress their function. NET degradation by DNASE1L3-expressing dendritic cells restores T-cell infiltration and enhances anti-PD-(L)1 efficacy, identifying NETs as therapeutically tractable targets (69). Therapeutic strategies are shifting from wholesale neutrophil depletion toward selective disruption of TAN recruitment (CXCR2 inhibitors), functional reprogramming, and NET-targeted approaches (65, 70).

##### 2.1.4.2 Regulatory B cells and tumor-associated inhibitory B cells

Bregs represent a distinct B-cell subset with potent immunosuppressive functions, differing from antibody-secreting effector B cells, and their core characteristic is to mediate immune tolerance by secreting inhibitory cytokines or expressing co-inhibitory molecules (71).

Single-cell studies have uncovered extensive B-cell heterogeneity in cancer. A pan-cancer atlas identified 20 B-cell subtypes, including tumor-associated atypical B cells (TAABs) linked to favorable immunotherapy outcomes (72, 73). Bregs suppress antitumor immunity via three core mechanisms: (a) secretion of IL-10, IL-35, and TGF-β to inhibit CD8^+^ T and NK cells; (b) surface expression of PD-L1, FasL, and TIM-1 to induce T cell apoptosis in a contact-dependent manner; and (c) exosomal transfer of inhibitory microRNAs to remotely reshape immune phenotypes.

Mechanistically, PPARδ has been identified as a key driver of tumor-induced IL-10^+^ Bregs; PPARδ inhibition enhances anti-PD-1 efficacy (74). In pancreatic cancer, B cells acquire an IL-35^+^ regulatory phenotype via synergistic BCR and TLR4 signaling, with protein kinase D2 (PKD2) as a critical downstream regulator. PKD2 inactivation blocks IL-35 production, reduces tumor growth, and restores T cell activity (75, 76). A renal cell carcinoma-specific CD27^‐^IgD^‐^ double-negative 1 (DN1) Breg subset was found within tertiary lymphoid structures, suppressing CD8^+^ T cells via IL-10 and TGF-β (7, 77).

##### 2.1.4.3 Subpopulations of immunosuppressive innate lymphoid cells

ILCs are a family of innate immune cells that lack rearranged antigen receptors and are widely distributed in mucosal and tumor tissues. Recent studies have revealed their remarkable functional heterogeneity, among which regulatory ILCs (ILCregs) and immunosuppressive type 2 innate lymphoid cells (ILC2s) are key suppressive subsets within the tumor microenvironment (TME) (78, 79).

ILCregs do not express Foxp3 and mediate immune tolerance through the secretion of IL-10 and TGF-β. Recent investigations have further clarified that intratumoral ILCregs highly express IDO1 and CD39, which deplete tryptophan and generate immunosuppressive adenosine via metabolic reprogramming, directly inhibiting the functions of effector T cells and NK cells (80). Immunosuppressive ILC2s promote tumor angiogenesis and epithelial-mesenchymal transition (EMT) while suppressing type 1 immune responses by secreting IL-4, IL-13, and thymic stromal lymphopoietin (TSLP) (78, 81).

A 2025 *Cellular & Molecular Immunology* study demonstrated that macrophages regulate PD-1 and CTLA-4 expression on ILC2s within the TME, maintaining ILC2s in a hypo-responsive state that prevents their exhaustion while preserving their suppressive potential (82). A 2024 *Nature* study first established that IL-33 activates lymphogenic ILC2s and drives the formation of tertiary lymphoid structures (TLS) in pancreatic cancer, with KLRG1 identified as a key surface marker of tumor-infiltrating ILC2s (83). Importantly, targeting KLRG1 on immunosuppressive ILC2s has emerged as a promising strategy to reverse their suppressive function. Abcuro, Inc. published proof-of-concept data describing KLRG1 as a promising new target for cancer immunotherapy, with clinical development of anti-KLRG1 antibodies currently underway (84).

##### 2.1.4.4 Unconventional immunosuppressive T cell subsets

Beyond conventional αβ T cells, several unconventional T cell subsets possess potent immunosuppressive functions, including γδ regulatory T cells (γδ Tregs), immunosuppressive MAIT cells, and CD4^+^CD8^+^ double-positive (DP) inhibitory T cells (85).

γδ T cells, normally innate-like effectors, acquire regulatory features in the tumor microenvironment. In pancreatic cancer, a subset expresses FoxP3, driven by mevalonate pathway intermediates (e.g., IPP) binding BTN3A1 in the presence of TGF-β and IL-15, correlating with poor outcomes (86, 87). Additionally, radiation-induced cGAS/STING signaling in macrophages recruits γδ T cells via CCL20, which then mediate MDSC recruitment and T cell suppression, driving radioresistance (88).

Immunosuppressive MAIT cells have been identified as novel regulators. In glioblastoma, MAIT cell signature genes correlate with poor survival and co-localize with TANs, forming a MAIT-TAN immunosuppressive axis (89). Their dual role in other cancers (colorectal, liver, lung) has also been documented (90).

CD4^+^CD8^+^ double-positive T cells were found to be 140-fold enriched in circulating tumor cell (CTC) clusters in advanced breast cancer. These DP T cells exhibit exhaustion and immune suppression, and their VLA-4 integrin binds tumor VCAM1, promoting CTC clustering and metastasis. Anti-VLA-4 antibodies block this interaction and inhibit metastasis in mouse models (91, 92).

##### 2.1.4.5 Tolerogenic dendritic cells and immunosuppressive plasmacytoid dendritic cells

Within the tumor microenvironment, tolerogenic DCs (tolDCs) and immunosuppressive plasmacytoid DCs (pDCs) lose immunostimulatory capacity and acquire tolerogenic functions (93). tolDCs express low levels of CD80/CD86 but high levels of IDO1, ARG1, and PD-L1, inducing T cell anergy and promoting Treg differentiation (94). In pancreatic cancer, an IDO1^+^ tolDC subset was identified in spleens (95). Tumor-derived extracellular vesicles trigger ER stress (PERK-ATF4, IRE1α-XBP1s) and PPAR-α-driven fatty acid oxidation, reprogramming DCs toward a pro-tolerogenic state (96).

**Figure 1:**
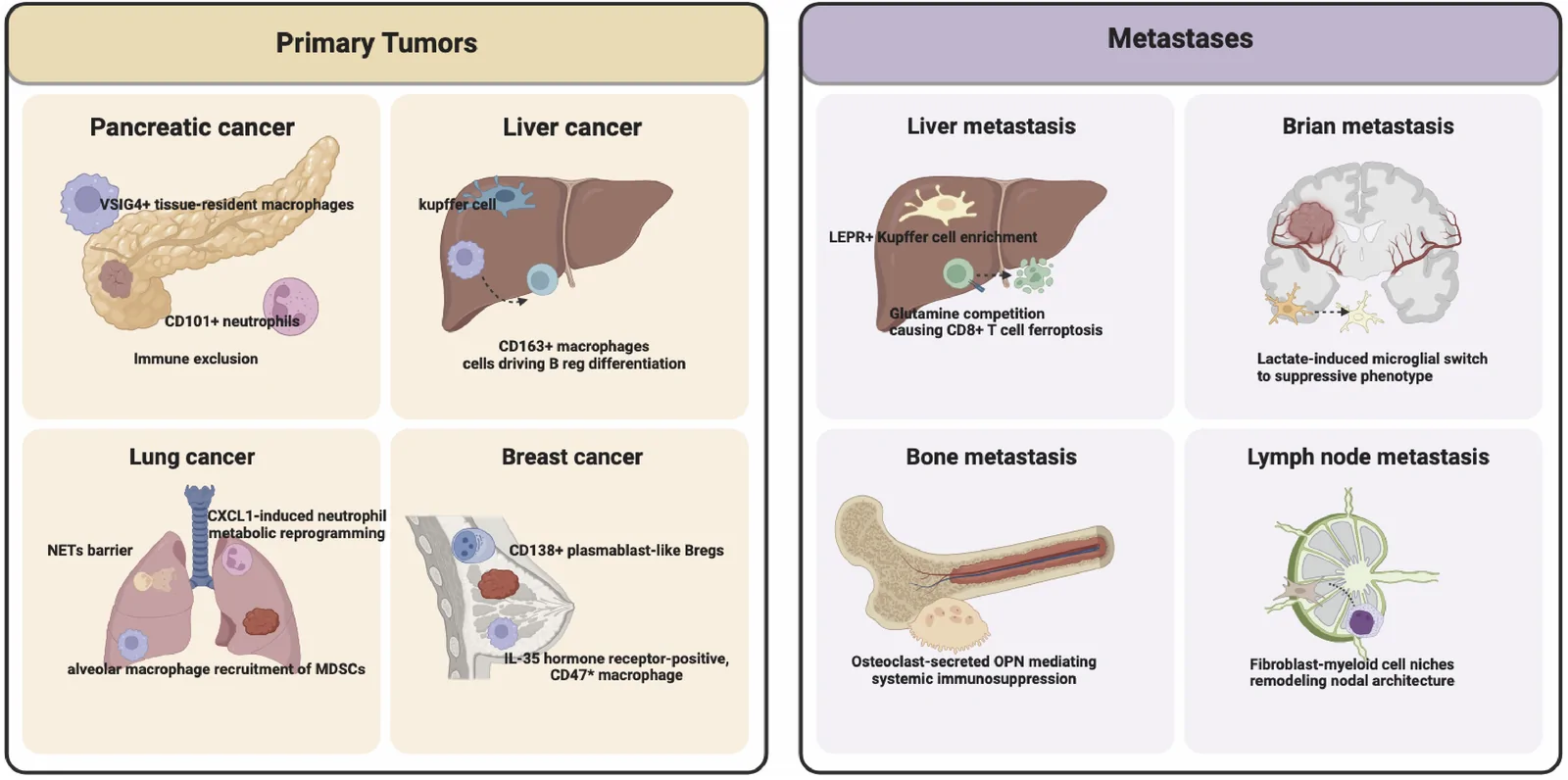
Functional diversity of immunosuppressive cells in the tumor microenvironment. Shown are the major suppressive cell types (MDSCs, TAMs, Tregs, TANs, Bregs, tolDCs, and ILCregs) and their key inhibitory mediators. These cells converge on common effector pathways—suppressing T cell proliferation, cytotoxic function, and inducing exhaustion—while cancer cells escape immune attack.

Immunosuppressive pDCs lose type I IFN secretion and instead upregulate the PPARγ-CPT1A-FAO axis, sustaining ICOSL expression and Treg generation; CPT1A inhibition restores CD8^+^ T cell function (97, 98). The C5a/C5aR1 axis promotes tolDC migration to lymph nodes, impairing antitumor immunity (99) (**Figure 1**).

### 2.2 Synergistic networks within the immunosuppressive microenvironment

#### 2.2.1 Organ-specific features of immunosuppressive microenvironments in primary tumors

The anatomical location of a tumor shapes its immunosuppressive architecture through three interacting layers: resident immune composition, metabolic/vascular conditions and stromal organization. Across pancreas, liver, lung and brain, a shared core logic is evident—myeloid and lymphoid suppressive programs reduce effector-cell function and access—yet the dominant barrier differs by organ. Pancreatic tumors are often constrained by dense desmoplasia; the liver builds on a tolerance-biased baseline; the lung couples NET-mediated exclusion with alveolar macrophage programs; and the brain adds blood–brain barrier and microglial constraints. This comparison explains why a single intervention is unlikely to transfer uniformly across organs, and why mechanism-matched combinations should be tailored to the local dominant barrier (100).

##### 2.2.1.1 Pancreas: stroma-dominated immune exclusion

Pancreatic ductal adenocarcinoma (PDAC) represents the archetype of a fibroinflammatory tumor, in which non-malignant stromal elements comprise the volumetric majority of the tumor tissue and form a uniquely dense physical barrier that severely restricts immune cell access (101, 102). Unlike tumors in other organs, PDAC is characterized by an extensive desmoplastic reaction driven by cancer-associated fibroblasts (CAFs) and a dense extracellular matrix, which together create an “immune desert” microenvironment largely devoid of effective T-cell infiltration. Within this stroma-rich niche, VSIG4^+^ tissue-resident macrophages have emerged as a core immunosuppressive subset that orchestrates local immune evasion through epigenetic modulation of SPP1, thereby recruiting neutrophils and suppressing antigen-specific immunity (103, 104). The reciprocal CAF-MDSC-TAM signaling axis sustains a self-reinforcing cycle of immunosuppression that renders PDAC largely refractory to current immunotherapies (101, 103).

##### 2.2.1.2 Liver: tolerance-hardwired immunosuppression

The liver possesses a uniquely tolerant immunological baseline, driven by its continuous exposure to gut-derived antigens. Tissue-resident Kupffer cells, together with liver sinusoidal endothelial cells and stellate cells, constitutively present antigens in a non-inflammatory context and secrete regulatory cytokines such as IL-10 and TGF-β, thereby dampening effector T-cell priming and promoting regulatory T cell (Treg) differentiation (103, 104). This tolerogenic programming--essential for maintaining systemic immune homeostasis--creates a permissive soil for tumor immune evasion. A recent groundbreaking study revealed a liver-specific immunosuppressive mechanism: hepatocellular carcinoma (HCC) cells secrete ammonia, which is metabolized by intratumoral Tregs via argininosuccinate lyase (ASL) and spermine synthase (SMS). Spermine directly interacts with PPARγ, enhancing oxidative phosphorylation and consolidating Treg suppressive function, thereby driving resistance to anti-PD-1 therapy (105). This ammonia-Treg metabolic adaptation represents a striking example of how the liver’s unique metabolic environment (the organ is the principal site for ammonia detoxification) is co-opted by tumor cells to reinforce local immunosuppression. Aberrant bile acid metabolism further activates hepatic stellate cells and induces Kupffer cell conversion toward a tolerogenic phenotype, collectively strengthening the liver’s baseline tolerance into a tumor-protective immunosuppressive network (106–108).

In HCC, tumor-intrinsic metabolic programs can directly shape this Treg-permissive niche. AARS1-mediated ATF6 lactylation activates TDO2 and the kynurenine pathway, forming a tumor–Treg metabolic feedback circuit that promotes Treg differentiation and immune evasion (105). Complementary single-cell and spatial analyses identify CD177+ tumor-infiltrating Tregs as a distinct, immunosuppressive population in HCC, while PSME3-driven glycolytic reprogramming promotes Treg accumulation and anti-PD-1 resistance (106, 109). These findings support a tissue-specific HCC framework, but require independent validation before biomarker-guided clinical use.

A complementary preclinical strategy targets Treg bioenergetics: puerarin binds MIC19, disrupts mitochondrial cristae organization and weakens tumor-infiltrating Treg oxidative metabolism, enhancing antitumor immunity in HCC models (110–112). This approach is promising but remains preclinical and should not be interpreted as established clinical efficacy.

##### 2.2.1.3 Lung: NET- and alveolar macrophage-driven immune exclusion

The lung microenvironment imposes distinct immunosuppressive features on both primary lung cancer and pulmonary metastases. Neutrophil extracellular traps (NETs) play a prominent role by forming physical barriers that impede effector T-cell infiltration and by promoting tumor growth. A study demonstrated that NETs stabilize m^6^A-mediated SLC2A3 mRNA, inducing ferroptosis resistance in lung adenocarcinoma cells and directly suppressing CD8^+^ T cell activity (113). Clinical evidence shows that high NET density in advanced NSCLC patients correlates with shorter progression-free survival (PFS: 8 vs 20 months) and overall survival (OS: 14.7 vs 29.8 months), and is inversely correlated with CD8^+^ T cell density (114).

Furthermore, tumor-associated AMs undergo a time-dependent metabolic shift toward glycolysis, resulting in impaired secretion of the tumor suppressor SOCS3--a phenotype that can be reversed by targeting glycolytic flux (115). Tumor-derived signals further skew these macrophages toward an M2-like phenotype, recruiting MDSCs through the CCL2-CCR2 axis and amplifying immunosuppressive cascades (116). The combination of NET-mediated physical exclusion and alveolar macrophage-driven suppression creates an aggressive immunosuppressive landscape that underlies the high rates of primary and acquired resistance to immune checkpoint inhibitors in lung cancer.

##### 2.2.1.4 Brain: immune-privileged niche with glial cell reprogramming

The central nervous system (CNS) is immunologically privileged, with the blood-brain barrier restricting immune cell trafficking. Tissue-resident microglia--the brain’s specialized macrophages--derive from yolk sac progenitors and are maintained independently of bone marrow input, endowing them with a transcriptional program distinct from other tissue macrophages (117). In the brain tumor microenvironment, microglia undergo profound reprogramming toward tumor-supportive phenotypes, losing their surveillance functions and secreting immunosuppressive factors such as TGF-β and IL-10 (118, 119).

A 2025 *Cancer Cell* study further demonstrated that activation of the Rela/NF-κB pathway in microglia promotes melanoma brain metastasis, and targeting this pathway elicits microglial reprogramming toward a pro-inflammatory phenotype, enhancing anti-tumor immunity and improving responses to immune checkpoint inhibitors (120). Additionally, single-cell multi-omics profiling of gliomas revealed four distinct immunomodulatory expression programs in myeloid cells, including microglial inflammatory and scavenger immunosuppressive programs unique to primary brain tumors, whose relative expression can predict immunotherapy response and overall survival (121). Growing evidence highlights the need for strategies that specifically reprogram microglial function rather than simply depleting myeloid populations in CNS tumors (119, 120).

#### 2.2.2 Core differences in immunosuppressive microenvironments between primary tumors and metastases

Metastatic lesions are not simple replicas of primary tumors; they undergo distinct immune adaptation within target organ microenvironments, often establishing more robust immunosuppressive networks. Recent spatial and single-cell studies have defined five key differences.

##### 2.2.2.1 Tissue-adaptive reprogramming of immunosuppressive cells

Metastases harbor organ-specific myeloid and lymphoid subsets that are rare in primary lesions. In liver metastases, chemotherapy reprograms Kupffer cells into a LEPR^+^ subset that suppresses antitumor immunity via MerTK-dependent efferocytosis (122). In brain metastases, single-cell spatial mapping revealed BZW2^+^ tumor cells that actively create immune-excluded “cold” niches, a feature absent in matched primaries (123).

##### 2.2.2.2 Divergent immunosuppressive mechanisms

Primary tumors rely mainly on local inhibitory factors, whereas metastases co-opt resident cells to build systemic suppressive networks. Bone metastases enhance osteoclast-derived osteopontin (OPN), which circulates to distant tumors and impairs CD8^+^ TCF1^+^ progenitor-exhausted T cells, driving systemic ICB resistance (124). In lymph node metastases, CAFs and immunomodulatory macrophages form organized niches that recruit ICOS^+^ Tregs and dismantle antigen-presenting compartments--a coordinated remodeling not seen in primary tumors (125).

##### 2.2.2.3 Metabolic microenvironment remodeling

Metabolic competition intensifies in metastases, especially in the liver. Glutamine shortage in hepatic metastases drives infiltration of GPR109A^+^ immunosuppressive myeloid cells via the IRE1α/XBP1 pathway, while glutamine depletion also induces CD8^+^ T cell ferroptosis--a vulnerability less prominent in primary tumors (126).

##### 2.2.2.4 Antigen presentation dysfunction

Metastases exhibit lower immunogenicity and more severe antigen-processing defects. In brain metastases, BZW2^+^ tumor cells inversely correlate with TAP1, suppressing MHC-I antigen presentation (123). Tolerogenic DCs (IDO1^+^, PD-L1^+^, low CD80/CD86) are enriched in metastatic sites, directly inducing T cell anergy (125).

##### 2.2.2.5 Extracellular matrix remodeling and stromal barriers

**Figure 2:**
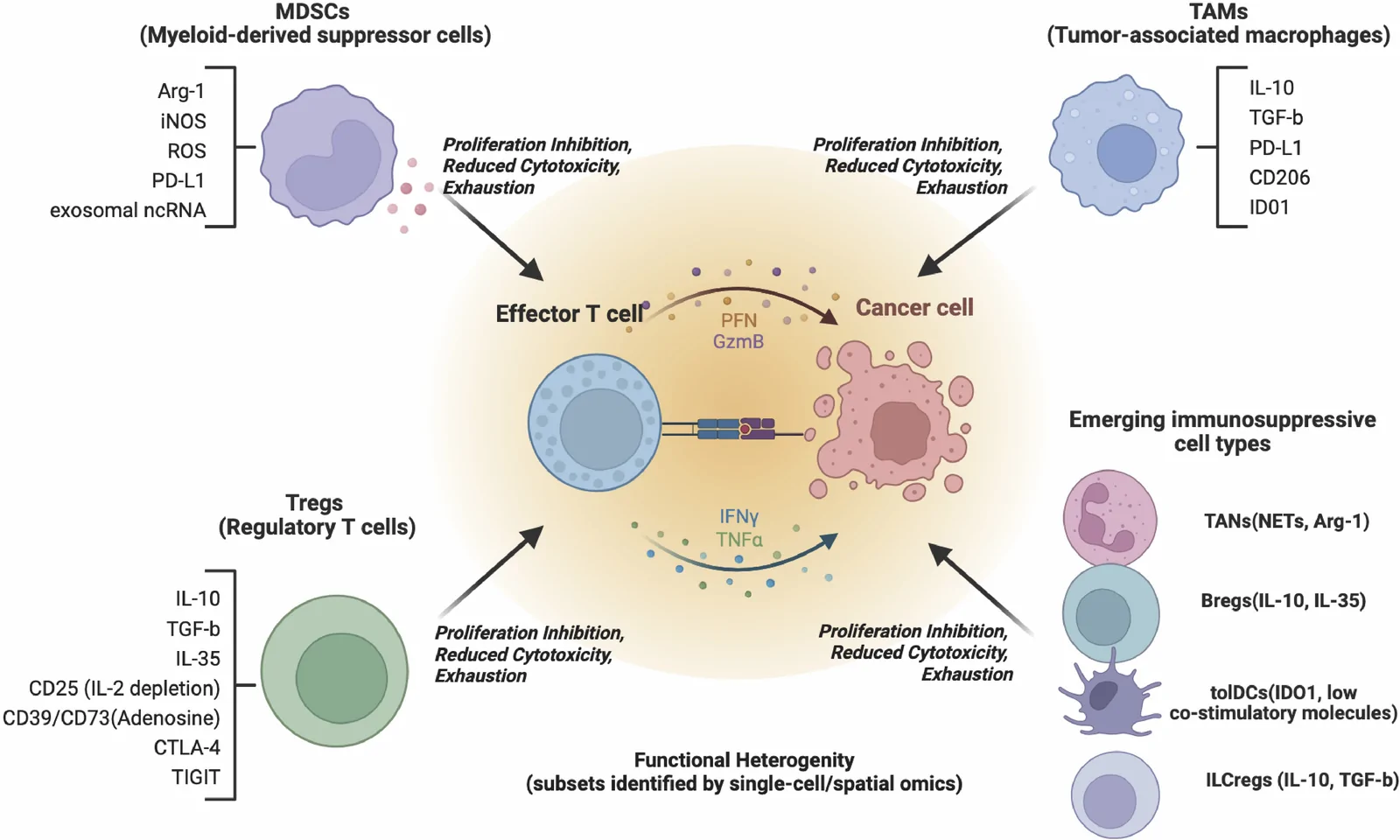
Primary tumor and metastatic immunosuppressive niches across organs. Left: primary tumors (pancreas, liver, lung, breast) display distinct dominant suppressive cell subsets and molecular drivers. Right: metastatic lesions undergo tissue-adaptive reprogramming, with organ-specific metabolic and stromal alterations that further amplify immunosuppression.

Metastases often develop denser physical barriers. In lymph node metastases, CAFs and immunomodulatory macrophages create spatially organized niches that exclude effector T cells. In breast cancer, a metastasis-specific TLR4-dependent fibroblast-monocyte axis recruits immunosuppressive monocytes via CCL2/CCL7, a stromal adaptation not typically observed in primary tumors (127) (**Figure 2**).

#### 2.2.3 Intercellular signaling crosstalk and interactions with non-immune cells

The immunosuppressive TME is best understood as a set of interconnected functional circuits rather than a sequential list of cell types. Here, we group crosstalk into three categories: (a) reciprocal immune-cell loops, such as TAM–Treg and NET–MDSC–Treg axes; (b) stromal and vascular hubs, in which CAFs and extracellular matrix organization regulate immune positioning and access; and (c) metabolite- and vesicle-mediated signals that coordinate local and systemic myeloid skewing. This framework highlights both the mechanism of suppression and the point at which a therapeutic intervention might break a self-reinforcing loop.

##### 2.2.3.1 Crosstalk among immunosuppressive cells

Tregs and TAMs reciprocally reinforce each other: Tregs promote M2-like TAM polarization through multiple mechanisms, while TAMs in turn facilitate Treg recruitment and activation (128). A recent study systematically dissected the metabolic interplay between TAMs and Tregs, revealing that metabolic changes in both cell types--regulated by signaling cascades and epigenetic reprogramming--significantly influence their immunosuppressive phenotypes and collaborative suppression of CD8^+^ T cells (129). Bregs also engage in cross-talk with Tregs. By secreting IL-35, Bregs effectively expand the Treg compartment, and Tregs reciprocally influence Breg function (130).

##### 2.2.3.2 Non-immune cells as hubs of immunosuppression

CAFs serve as central orchestrators of the immunosuppressive network. Using multiple tumor models and patient biopsies, Varveri et al. demonstrated that α-SMA^+^ CAFs can form immunological synapses with Foxp3^+^ Tregs. These CAFs phagocytose and process tumor antigens, exhibit a tolerogenic phenotype, and instruct Treg activation and proliferation in an antigen-specific and autophagy-dependent manner (131). Beyond local TME, tumor cells can remotely reprogram bone marrow hematopoiesis via exosomes and cytokines, driving the generation of immunosuppressive myeloid cells and establishing systemic immune suppression (132).

##### 2.2.3.3 Key multicellular suppressive axes

NETs function as molecular bridges that link distinct immunosuppressive populations. A study in *Cancer Cell* demonstrated that NETs activate MDSCs via TLR9 signaling (upregulating Arg-1 and iNOS) and promote Treg differentiation, thereby establishing a NETs-MDSCs-Tregs cascade; targeting this axis significantly enhances anti-PD-1 efficacy (133) Additionally, tumor-derived metabolites—including adenosine, lactate, and kynurenine--act as paracrine signals that simultaneously suppress effector T cells and expand Tregs and MDSCs (134). Adenosine, in particular, acts through A_2_A receptors to inhibit effector T cell function while promoting Treg expansion (135). Collectively, these interconnected signaling axes create a self-reinforcing immunosuppressive ecosystem that poses a major barrier to effective cancer immunotherapy.

**Figure 3:**
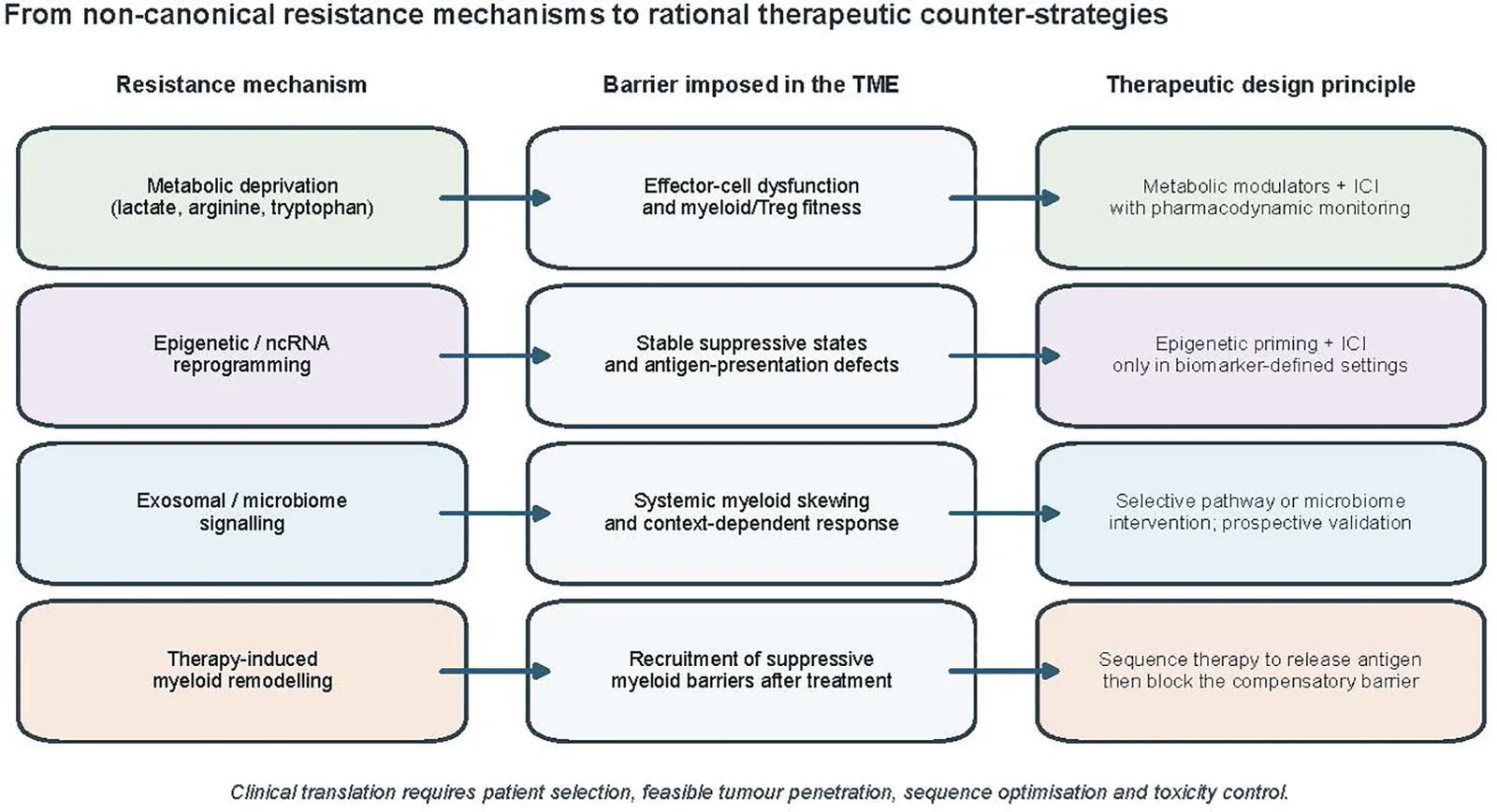
From non-canonical resistance mechanisms to rational therapeutic counter-strategies. Metabolic, epigenetic/non-coding RNA, exosomal/microbiome and therapy-induced regulatory layers create distinct suppressive barriers. Translation requires mechanism-matched intervention, patient selection, feasible tumor penetration, treatment-sequence optimization and toxicity control.

The therapeutic relevance of these networks depends on evidence strength. Mechanistic findings from model systems may define actionable hypotheses, whereas biomarker associations in patient cohorts should be interpreted as context-dependent until prospectively validated. Below, the major non-canonical regulatory layers are linked to the barriers they impose and to the corresponding design principles for combination therapy (**Figure 3**).

## 3 Regulatory layers of therapy resistance beyond canonical pathways

Immunotherapy resistance arises from a multidimensional network beyond canonical immune checkpoint signaling, encompassing metabolic reprogramming, epigenetic regulation, microbiome crosstalk and therapy-induced adaptive remodeling of immunosuppressive cells (134, 135). The strength of evidence is not uniform across these layers. Direct perturbation studies in relevant models provide mechanistic support; correlative patient datasets may identify clinically important associations but do not establish causality; and microbiome, exosomal and some epigenetic observations remain strongly context-, assay- and treatment-dependent (136). We use this distinction to frame the following sections and to avoid presenting preclinical promise as clinical validation.

### 3.1 Metabolic–immune checkpoint crosstalk

Metabolic reprogramming and immune checkpoints form a bidirectional regulatory network that drives resistance (137).

#### 3.1.1 FATP2-PPARγ-PD-L1 axis

Fatty acid transport protein 2 (FATP2) is highly expressed in MDSCs, where it mediates long-chain fatty acid uptake, activates PPARγ, and upregulates PD-L1 expression. This axis is hyperactivated in pancreatic and non-small cell lung cancers and correlates with anti-PD-1 resistance. Targeting FATP2 reduces MDSC PD-L1 levels and restores CD8^+^ T cell cytotoxicity (138, 139).

#### 3.1.2 CTLA-4-mediated glycolysis inhibition in APCs

Beyond competing for CD80/CD86, CTLA-4 on Tregs suppresses antigen-presenting cell (APC) function by inhibiting glycolysis. CTLA-4 ligation triggers AMPK signaling, downregulates the glycolytic enzyme HK2, and impairs APC costimulation, thereby exacerbating local immunosuppression (140).

### 3.2 Epigenetic and non-coding RNA regulation

Epigenetic modifications and non-coding RNAs sustain the functional stability of immunosuppressive cells (141).

#### 3.2.1 HDAC6 maintains MDSC suppressive activity

Histone deacetylase 6 (HDAC6) is highly expressed in MDSCs and mediates H3K9 deacetylation to sustain arginase-1 (Arg-1) expression. HDAC6 inhibitors downregulate Arg-1, drive MDSC conversion toward an M1-like phenotype, and improve anti-PD-1 efficacy (142).

#### 3.2.2 miR-155–SOCS1–STAT3 axis in TAMs

miR-155 is enriched in TAMs and promotes M2 polarization by silencing SOCS1, thereby activating STAT3 signaling. miR-155 inhibition reduces M2-like TAM abundance and restores antitumor immunity (143).

#### 3.2.3 STAT1–IFITM3 negative feedback loop in Tregs

An intrinsic IFN-γ-STAT1-IFITM3 axis preserves Treg functional homeostasis. IFN-γ induces STAT1 phosphorylation and IFITM3 transcription; IFITM3 then binds JAK1/2 to limit STAT1 activation, forming a self-limiting loop. Disrupting this loop drives Treg fragility (reduced FOXP3, increased IFN-γ) and enhances antitumor immunity, offering a strategy to selectively target tumor-resident Tregs (144).

### 3.3 Distant regulation by the microbiome

Commensal microbes remotely shape the tumor immune microenvironment through metabolites and structural components (145).

#### 3.3.1 SCFAs promote Treg differentiation

Short-chain fatty acids (acetate, propionate, butyrate) from dietary fiber fermentation enhance peripheral Treg differentiation via GPR43 activation and HDAC inhibition. Reduced SCFA-producing bacteria in hepatocellular carcinoma patients correlates with diminished Treg induction; butyrate supplementation restores this effect (146).

#### 3.3.2 Porphyromonas gingivalis expands MDSCs

The oral pathogen *P. gingivalis* releases LPS, triggering TLR4 signaling that drives bone marrow hematopoiesis toward the MDSC lineage. *P. gingivalis* infection correlates with anti-PD-1 resistance in colorectal cancer; pathogen eradication reduces systemic MDSCs and restores immunotherapy sensitivity (147).

### 3.4 Therapy-induced adaptive remodeling of immune cells

Chemotherapy and radiotherapy trigger adaptive changes in immunosuppressive cells that reinforce resistance (148).

#### 3.4.1 Chemotherapy upregulates CD47 on MDSCs

Gemcitabine and other agents induce CD47 expression on MDSCs. CD47 engagement with macrophage SIRPα suppresses phagocytosis, allowing MDSCs to evade immune clearance. Combining chemotherapy with CD47 blockade eliminates MDSCs and restores antitumor immunity (149).

#### 3.4.2 Radiotherapy induces VEGF secretion by TAMs

Ionizing radiation stimulates TAMs to secrete VEGF, which activates VEGFR2-AKT signaling in tumor cells, enhancing DNA repair and conferring radioresistance. Concurrent VEGF inhibition improves radiotherapy efficacy (150).

## 4 From systematic synergistic strategies to clinical translation

Single-agent ICIs only confer clinical benefits to 20%-40% of patients with “hot tumors”, while exhibiting limited efficacy in “cold tumors” such as pancreatic cancer and glioblastoma. The primary obstacle lies in the multidimensional drug resistance barrier constructed by the immunosuppressive tumor microenvironment (151, 152). In recent years, cancer immunotherapy has evolved from monotherapy into the era of systematic synergistic intervention. By integrating multi-pathway immune blockade, targeted therapy, conventional radiotherapy and chemotherapy, metabolic modulation, microbiome intervention and cellular therapy, comprehensive remodeling of the immunosuppressive network can be achieved. A growing number of phase III clinical trials have yielded landmark advances, markedly improving long-term survival outcomes in patients with solid tumors (153, 154).

A practical “cold-to-hot” sequence is therefore required. First, the dominant myeloid, stromal or vascular barrier must be reduced or reprogrammed to permit antigen release and chemokine remodeling. Second, antigen-presenting cells and permissive chemokine gradients must support entry of cytotoxic T cells into tumor nests. Third, checkpoint and metabolic constraints must be relieved so that infiltrating clones expand, retain cytotoxic function and persist. Failure at any stage can leave a tumor inflamed at the margin but functionally excluded at the tumor core; this is why combination design should be based on the barrier sequence rather than on nonspecific drug stacking.

### 4.1 Rationale and design of synergistic strategies to overcome immunosuppressive barriers

Clinical translation requires a parallel feasibility filter. Across dual checkpoint blockade, ADC–ICI combinations, metabolic targeting, microbiome interventions and engineered-cell platforms, the central questions are whether the relevant target is present in the intended patient subset, whether the agent can penetrate a dense tumor and reach the relevant compartment, whether treatment sequencing avoids compensatory myeloid recruitment, and whether added activity justifies systemic or organ-specific toxicity. Tumor and target-expression heterogeneity can make a mechanistically attractive combination ineffective or unsafe outside a biomarker-defined setting.

#### 4.1.1 Multi-pathway synergistic blockade of immune checkpoint inhibitors

The rationale for combining ICIs targeting distinct pathways has evolved from simple pathway complementarity to a mechanistic understanding of clonal dynamics, circadian regulation, and multi-specific molecular design.

##### 4.1.1.1 Clonal turnover and temporal synergy

Classical studies established that the PD-1/PD-L1 and CTLA-4 pathways regulate T cell responses at distinct phases: CTLA-4 primarily controls the priming phase within lymph nodes, whereas PD-1 acts at the effector phase in peripheral tissues. However, recent advances using single-cell RNA sequencing and T cell receptor (TCR) lineage tracing have fundamentally revised this model. Combination therapy does not merely maintain the activation of the same T cell clones; instead, it induces sequential waves of independent clonal responses. Anti-CTLA-4 antibodies expand the pool of progenitor-exhausted T cells in secondary lymphoid organs, while anti-PD-1 antibodies convert these reserve cells into cytotoxic effectors that infiltrate the tumor. This dynamic clonal turnover creates a temporal window of synergy, explaining why appropriately sequenced dual blockade yields more durable antitumor immunity than monotherapy (155).

##### 4.1.1.2 Circadian regulation of the tumor-immune interface

The tumor microenvironment exhibits intrinsic circadian oscillations. The infiltration capacity and effector function of CD8^+^ T cells fluctuate throughout the day, a rhythm driven by the biological clock of tumor vascular endothelial cells. Mathematical modeling has demonstrated that the efficacy of immunotherapy pulses is tightly coupled to their alignment with peak T cell infiltration times. Synchronizing ICI administration with these endogenous rhythms maximizes antitumor effects while minimizing systemic toxicity, providing a rationale for chrono-immunotherapy strategies (155, 156).

##### 4.1.1.4 Bispecific and multi-specific antibody platforms

An in-depth understanding of cross-regulation among immunosuppressive networks has enabled the design of single agents that simultaneously block multiple checkpoints or co-target stromal barriers. PD-L1/TGF-β bispecific antibodies are designed to co-engage cancer-associated fibroblasts (CAFs) that co-express both molecules. This dual targeting downregulates collagen synthesis and stromal remodeling genes, dismantling the physical barrier that excludes T cells, while simultaneously promoting dendritic cell maturation and antigen presentation. The result is a positive feedback loop of “stromal remodeling–immune activation” that cannot be achieved by either monotherapy or simple combination of separate antibodies (157, 158).

Similarly, PD-1/CD47 bispecific antibodies bridge adaptive and innate immunity. By blocking T cell inhibitory signaling (PD-1/PD-L1) and abrogating the “don’t eat me” signal on macrophages (CD47/SIRPα), these agents synergistically activate T cell-mediated killing and macrophage phagocytosis. This design exploits the complementary roles of the two arms of the immune system, offering a mechanism to overcome resistance that arises from sole reliance on T cell checkpoint blockade (159).

#### 4.1.2 Mechanistic complementarity and synergy between immunotherapy and antibody-drug conjugates

The combination of ADCs with ICIs operates through deep mechanistic complementarity at the molecular and cellular levels, extending well beyond simple additive cytotoxicity. Early studies regarded ADCs solely as targeted chemotherapeutic agents; however, recent advances in basic immunology have revealed their potent immunomodulatory potential. Beyond precise tumor cell killing, ADCs can systematically remodel the tumor TME, converting immunologically “cold” tumors into “hot” tumors and thereby creating permissive conditions for ICI efficacy (158).

##### 4.1.2.1 Immunogenic cell death as the core foundation

Immunogenic cell death (ICD) constitutes the primary mechanism underlying ADC-ICI synergy. Using single-cell transcriptomics and proteomics, recent studies have systematically dissected how different ADC payloads induce ICD through distinct molecular pathways. Topoisomerase I inhibitor payloads (e.g., DXd, SN-38) activate the endoplasmic reticulum stress pathway (PERK–eIF2α–ATF4), leading to significant upregulation of calreticulin (CRT) on the tumor cell surface, along with the release of high-mobility group box 1 (HMGB1) and adenosine triphosphate (ATP). These damage-associated molecular patterns (DAMPs) bind to pattern recognition receptors (PRRs) on DCs, triggering DC maturation and enhancing antigen cross-presentation. In contrast, microtubule inhibitor payloads (e.g., MMAE, MMAF) induce substantially weaker ICD activity. This differential immunogenicity provides a rational basis for prioritizing ADC payloads in combination with ICIs (160, 161).

##### 4.1.2.2 Multipronged remodeling of the tumor immune microenvironment

Beyond ICD induction, ADCs reshape the TME through several complementary mechanisms. They can specifically deplete immunosuppressive myeloid populations--including MDSCs and M2-polarized TAMs--that express the target antigen. Simultaneously, ADCs promote the repolarization of TAMs toward an M1-like phenotype, normalize aberrant tumor vasculature to improve effector T cell infiltration and drug delivery, and release abundant tumor-associated antigens (TAAs) and neoantigens that facilitate DC cross-presentation (162–164).

##### 4.1.2.3 Bidirectional cross-regulation with immune checkpoints

Basic research has uncovered a complex, bidirectional relationship between ADCs and ICIs. ADC-induced IFN-γ upregulates PD-L1 expression on tumor cells--a phenomenon initially interpreted as an immunosuppressive side effect. However, recent studies demonstrate that this ADC-driven PD-L1 upregulation precisely creates a therapeutic vulnerability, providing an enhanced target for subsequent PD-1/PD-L1 blockade. This establishes a rational sequence of “ADC-induced PD-L1 expression followed by ICI-mediated checkpoint blockade.” Concurrently, ADCs downregulate other immunosuppressive molecules on tumor cells, including TGF-β and VEGF, further alleviating the immunosuppressive milieu (165).

##### 4.1.2.4 Optimized combination principles derived from mechanism

Building on these mechanistic insights, several design principles have emerged to maximize synergy. These include: a. prioritizing ADCs with topoisomerase I inhibitor payloads that exhibit strong ICD-inducing capacity; b. employing sequential administration (“ADC first, ICI second”) to allow for DAMP release, DC activation, and T cell priming before checkpoint blockade; and c. developing bispecific ADCs that concurrently target tumor antigens and immunosuppressive pathways, thereby improving specificity and amplifying ICD. Collectively, these principles provide a robust mechanistic framework for the rational design of ADC-ICI combination regimens (166, 167).

#### 4.1.3 Temporal and spatial optimization of synergy between immunotherapy and conventional therapies

The synergy between immunotherapy and conventional chemotherapy or radiotherapy has evolved from empirical co-administration to mechanistically guided optimization of timing, dosing, and spatial targeting. Traditional cytotoxic therapies do not simply kill tumor cells; they reshape the TME through distinct immunomodulatory pathways that exhibit strict temporal, dose-fractionation, and spatial dependencies (168).

##### 4.1.3.1 Chemotherapy: the “immunotherapeutic window” and metronomic scheduling

The immunomodulatory effects of chemotherapy follow a tight temporal sequence. Early concurrent administration regimens were often suboptimal because chemotherapy non-selectively depleted cycling immune cells. In contrast, preclinical studies have defined a transient “immunotherapeutic window” that opens shortly after chemotherapy. During this window, tumor cells undergoing ICD release DAMPs and tumor antigens, DCs mature and initiate cross-presentation, and effector T cells begin clonal expansion. Administration of ICIs within this window maximizes synergistic antitumor activity, whereas premature or delayed dosing attenuates efficacy (169). The optimal timing is further dictated by the immunogenicity of individual chemotherapeutic agents: strong ICD inducers (e.g., anthracyclines, topoisomerase I inhibitors) require a sequential “chemotherapy-first, ICI-second” regimen, while less immunogenic agents may be compatible with concurrent scheduling.

Low-dose, high-frequency metronomic chemotherapy offers a distinct mechanistic advantage. Preclinical evidence demonstrates that metronomic cyclophosphamide selectively depletes Tregs within the TME. The molecular basis for this selectivity lies in the high expression of aldehyde dehydrogenase 1A1 (ALDH1A1) by intratumoral Tregs, which metabolizes cyclophosphamide to its more toxic derivative 4-hydroxycyclophosphamide (4-HC). Effector T cells, lacking this enzyme, are relatively spared. Beyond Treg depletion, metronomic chemotherapy promotes DC maturation, enhances NK cell cytotoxicity, and inhibits tumor angiogenesis--collectively remodeling the immunosuppressive TME through multiple parallel mechanisms (170, 171).

##### 4.1.3.2 Radiotherapy: dose fractionation, the Cgas-STING pathway, and myeloid-mediated resistance

The synergy between radiotherapy and immunotherapy depends critically on dose fractionation rather than total dose alone. Different radiation regimens exert bidirectional effects on the immune microenvironment:

- Low-dose radiotherapy (≤2 Gy/fraction) promotes tumor vascular normalization by downregulating adhesion molecules on endothelial cells, thereby increasing effector T cell infiltration and drug delivery.
- Hypofractionated radiotherapy (≥5–8 Gy/fraction) induces DNA double-strand breaks, activating the cytoplasmic cGAS-STING pathway and producing abundant type I interferons. This effectively triggers ICD and initiates systemic antitumor immune responses.
- Ultra-high-dose radiotherapy (≥10 Gy/fraction) paradoxically attenuates immunogenicity by inducing the DNA exonuclease Trex1, which degrades cytoplasmic DNA and inhibits STING activation. This finding explains why hypofractionated, but not single-high-dose, regimens are optimally immunogenic (172).

Radiotherapy also engages immunosuppressive feedback loops that limit its efficacy. Recent studies have identified a key resistance mechanism: radiotherapy induces tumor cells to secrete the chemokine CXCL13, which recruits bone marrow-derived CXCR5^+^ monocytes into the TME. These recruited cells differentiate into immunosuppressive M2-polarized TAMs and MDSCs, which suppress CD8^+^ T cell function through IL-10, TGF-β, and PD-L1 expression. This myeloid-mediated negative feedback explains why radiotherapy alone rarely produces durable systemic immunity and provides a mechanistic rationale for combining radiotherapy with agents that block monocyte recruitment or myeloid suppression (173).

##### 4.1.3.3 The abscopal effect and *in situ* vaccination

Local radiotherapy can induce regression of distant, unirradiated tumors--a phenomenon termed the abscopal effect. Mechanistically, radiation-induced ICD releases tumor neoantigens that are cross-presented by DCs, priming tumor-specific T cells that subsequently travel to distant sites. However, the abscopal effect is rarely observed with radiotherapy alone due to concomitant activation of immunosuppressive myeloid cells. Understanding the spatiotemporal dynamics of antigen release, DC activation, and myeloid suppression has enabled the rational design of combination strategies that preserve the “*in situ* vaccine” effect while neutralizing the barriers that limit its systemic propagation (174, 175).

Together, these mechanistic principles-chemotherapy’s immunotherapeutic window, metronomic Treg depletion, fractionation-dependent STING activation, and radiotherapy-induced myeloid feedback loops-provide a framework for optimizing the timing, dose, and spatial delivery of conventional therapies to maximize synergy with immunotherapy.

#### 4.1.4 Cross-dimensional synergistic modulation of metabolism, immunity and microbiota

Metabolic reprogramming, immune dysfunction, and microbiota dysbiosis are not independent phenomena but constitute a highly interconnected, mutually reinforcing network within the TME. Understanding this cross-dimensional regulatory network has overcome the limitations of single-target strategies, establishing a mechanistic foundation for reversing immune checkpoint inhibitor resistance.

##### 4.1.4.1 Metabolic checkpoints that drive immunosuppression

Tumor-derived metabolic disturbances actively shape the immunosuppressive landscape. Fatty acid transport protein 2 (FATP2) is selectively upregulated in MDSCs. By mediating the uptake of long-chain fatty acids, FATP2 activates peroxisome proliferator-activated receptor γ (PPARγ) signaling, which in turn upregulates PD-L1 expression and stimulates prostaglandin E_2_ synthesis. This cascade creates a positive autocrine and paracrine feedback loop that amplifies MDSC suppressive potency. Preclinical models have shown that genetic or pharmacological interruption of the FATP2–PPARγ–PD-L1 axis restores the tumoricidal capacity of CD8^+^ T lymphocytes (176).

Lactate accumulation, a hallmark of dysregulated glycolysis in the TME, impairs effector T cell function through microenvironmental acidification and direct metabolic inhibition. Mechanistically, lactate suppresses TCR-induced nuclear translocation of NFAT, reducing interferon-γ and granzyme B production. In animal models, reducing intratumoral lactate concentration--either via lactate dehydrogenase (LDH) inhibition or by promoting lactate export--facilitates the differentiation of CD8^+^ T cells into stem cell-like memory phenotypes, thereby sustaining antitumor immunity (134, 177). These findings identify lactate and the LDH axis as actionable metabolic checkpoints.

##### 4.1.4.2 Microbiota-derived metabolites as remote regulators of immune cells

Host immune responses are substantially modulated by commensal microbiota through their metabolic products. Specific intestinal flora generate short-chain fatty acids (SCFAs; primarily acetate, propionate, and butyrate), secondary bile acids, and tryptophan metabolites. SCFAs promote peripheral Treg differentiation and functional maturation via G protein-coupled receptor 43 (GPR43) activation and histone deacetylase (HDAC) inhibition. In preclinical models, butyrate supplementation elevates Treg proportions in liver and intestinal tissues, contributing to local immunosuppressive homeostasis (178, 179). Other microbial metabolites, such as tryptophan catabolites (e.g., kynurenine), activate the aryl hydrocarbon receptor (AhR) on dendritic cells and T cells, further modulating the balance between effector and regulatory responses.

##### 4.1.4.3 Oral–gut axis and systemic MDSC expansion

Beyond the intestinal lumen, oral pathogens can remotely influence the TME through the oral–gut axis. *Porphyromonas gingivalis*, a keystone pathogen in periodontitis, releases lipopolysaccharide (LPS) that triggers Toll-like receptor 4 (TLR4) signaling in bone marrow hematopoietic stem cells. This signal redirects myeloid differentiation toward the MDSC lineage, leading to systemic accumulation of MDSCs in peripheral blood and distant tumor tissues. Preclinical studies have demonstrated that eradication of *P. gingivalis* or blockade of TLR4 signaling reduces MDSC abundance and restores sensitivity to immune checkpoint blockade (180, 181). This mechanism reveals a previously unrecognized link between oral dysbiosis and systemic immunosuppression.

##### 4.1.4.4 Integration of metabolic and microbial modulation

The convergence of metabolic and microbial pathways suggests rational combination strategies. For example, targeting FATP2 or LDH can be complemented by SCFA supplementation or microbiota remodeling to simultaneously reduce MDSC activity and optimize Treg-effector T cell balance. Such cross-dimensional approaches aim to reset the metabolic and microbial equilibrium of the host, thereby converting an immunosuppressive TME into one that supports durable antitumor immunity (139).

#### 4.1.5 Synergistic enhancement of cell therapy and immune checkpoint blockade

The combination of adoptive cell therapy (ACT) with ICIs addresses a fundamental limitation of each modality when used alone. ACT provides a large pool of tumor-reactive T cells, but these cells rapidly upregulate inhibitory receptors upon transfer and face a profoundly immunosuppressive TME. ICIs can relieve these brakes, but they require the presence of endogenous or transferred tumor-specific T cells. The mechanistic complementarity between ACT and ICIs has driven the rational design of engineered cell products and combination schedules that maximize *in vivo* persistence, function, and tumor infiltration.

##### 4.1.5.1 Mechanisms of ACT exhaustion and ICI-mediated rescue

Following adoptive transfer, both CAR-T cells and tumor-infiltrating lymphocytes (TILs) upregulate multiple immune checkpoint molecules, most notably PD-1, LAG-3, and TIM-3. This upregulation is driven by chronic antigen stimulation and inflammatory signals within the TME. PD-1 engagement by PD-L1 on tumor cells or antigen-presenting cells triggers SHP-1/2 phosphatases, which dephosphorylate key signaling molecules downstream of the TCR or CAR (e.g., ZAP70, PI3K), leading to reduced proliferation, cytokine production, and cytolytic activity. ICIs targeting the PD-1/PD-L1 axis directly reverse this exhaustion program in transferred cells, restoring their effector functions. Preclinical models have demonstrated that combining ACT with PD-1 or CTLA-4 blockade significantly enhances the persistence and antitumor activity of transferred T cells compared to either treatment alone (182).

##### 4.1.5.2 Gene editing to confer intrinsic checkpoint resistance

Rather than relying on exogenous ICIs, CAR-T cells can be engineered to be intrinsically resistant to checkpoint-mediated suppression. CRISPR/Cas9-mediated knockout of PD-1 in CAR-T cells has been shown to sustain effector function in the face of PD-L1-expressing tumors. Similarly, dual knockouts of PD-1 and other inhibitory receptors (e.g., LAG-3, TIM-3) further enhance resistance to exhaustion. These edited cells maintain higher levels of granzyme B, perforin, and pro-inflammatory cytokines upon repetitive antigen stimulation. The mechanistic advantage lies in uncoupling T cell activation from the negative feedback loop that normally limits excessive immune responses (183).

##### 4.1.5.3 Armored CAR designs that recruit endogenous immunity

Beyond passive resistance to suppression, “armored” CAR-T cells are engineered to secrete immunostimulatory cytokines that remodel the TME and recruit endogenous immune cells. CAR-T cells secreting IL-18, for example, not only enhance their own proliferation and persistence but also activate host NK cells and conventional CD8^+^ T cells. IL-18 acts on both adaptive and innate compartments, promoting a coordinated antitumor response that extends beyond the CAR-targeted antigen. Similarly, CAR-T cells secreting IL-12 or IL-15 have been developed, although with careful regulation to avoid systemic toxicity. These armored designs create a positive feedback loop: CAR-T cells kill tumor cells, release antigens, and activate endogenous immunity, which in turn helps control antigen-negative tumor variants (184).

##### 4.1.5.4 Off-the-shelf platforms with innate immune properties

Allogeneic, “off-the-shelf” cell therapies circumvent the logistical challenges of autologous products and can be engineered to possess intrinsic checkpoint resistance and innate antitumor activity. CAR-natural killer T (CAR-NKT) cells combine the antigen-specific cytotoxicity of T cells with the MHC-independent recognition of NKT cells. Because NKT cells do not require HLA matching and are less prone to cause graft-versus-host disease (GVHD), they are attractive candidates for standardized manufacturing. Importantly, CAR-NKT cells exhibit lower baseline expression of checkpoint molecules and are less susceptible to PD-1-mediated exhaustion compared to conventional CAR-T cells. This intrinsic resistance, combined with their rapid cytotoxic activity, provides a mechanistic rationale for combining CAR-NKT with ICIs or using them as standalone allogeneic products (185, 186).

Similarly, γδ T cells possess innate-like properties, including TCR-independent recognition of stress ligands and phosphoantigens. Gene editing to disrupt the endogenous TCRα/β chain eliminates the risk of GVHD while retaining the γδ TCR’s innate tumor recognition capability. Knockout of PD-1 further enhances their antitumor activity. Preclinical studies have shown that allogeneic γδ T cells, engineered with CARs or bispecific engagers, effectively control solid tumors and synergize with PD-1/PD-L1 blockade (187, 188).

##### 4.1.5.5 TIL therapy and checkpoint blockade synergy

TILs are naturally enriched for tumor-reactive clones but become exhausted during *in vitro* expansion and upon reinfusion. The combination of TIL therapy with ICIs addresses this by reversing the exhausted phenotype. Mechanistic studies indicate that PD-1 blockade during TIL expansion or immediately after infusion preserves the stem-like memory phenotype (TCF-1^+^, TIM-3^‐^) of TILs, which is essential for sustained antitumor activity. Moreover, ICIs can enhance the expansion of TILs *in vivo* by removing suppression from Tregs and MDSCs within the TME (189).

##### 4.1.5.6 Synthetic biology approaches for localized checkpoint modulation

**Figure 4:**
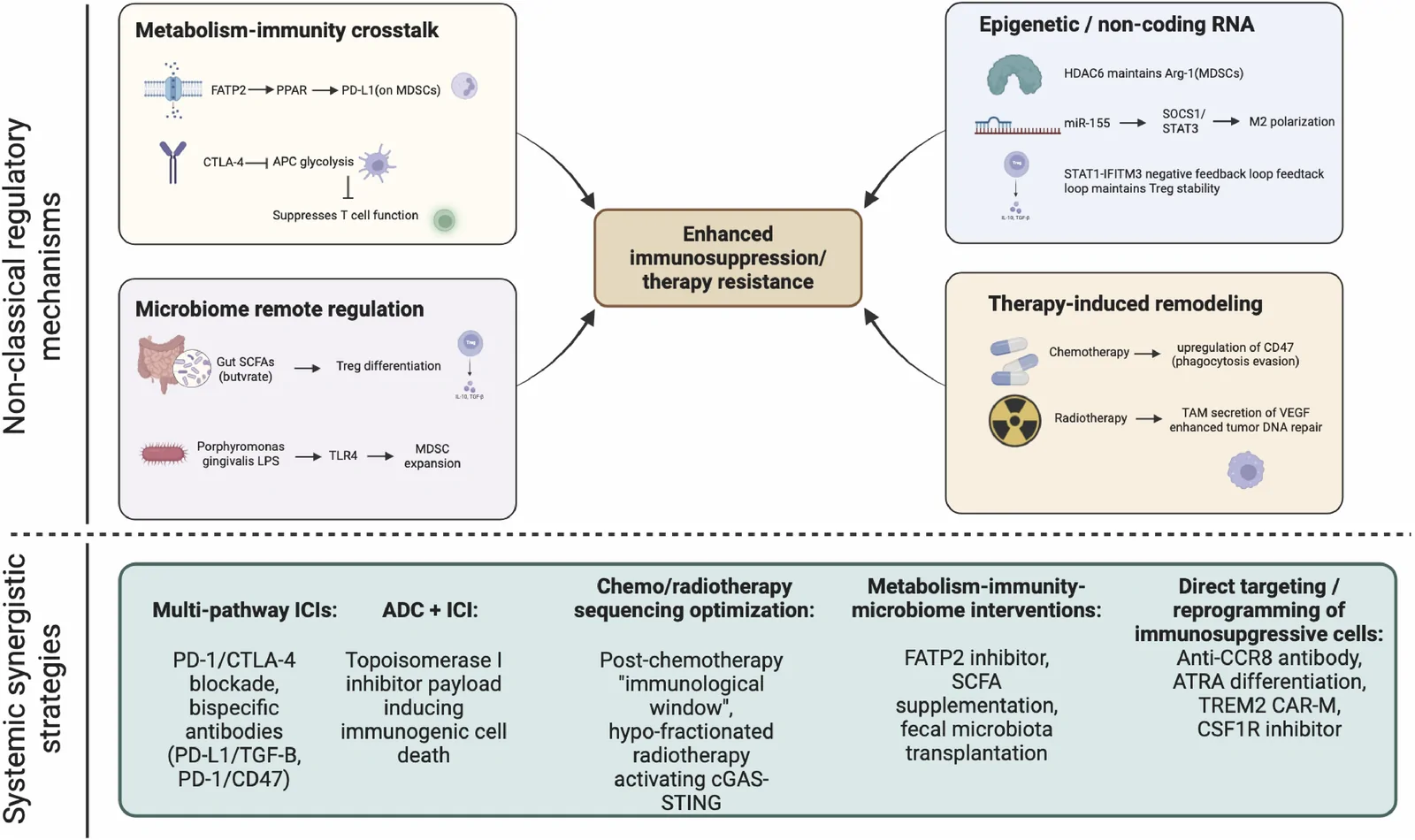
Integrating non-canonical resistance mechanisms with rational combination strategies. The upper panel outlines four non-canonical regulatory layers: metabolic–immune crosstalk, epigenetic regulation, microbiome modulation, and therapy-induced remodeling. The lower panel presents corresponding synergistic strategies: multi-pathway ICIs, ADC–ICI combinations, spatiotemporally optimized therapy, metabolism–immunity–microbiota interventions, and direct targeting of suppressive cells. This framework illustrates the paradigm shift from single-agent blockade to systematic synergy.

Emerging strategies aim to deliver checkpoint blockade directly to the TME via engineered cells. For example, CAR-T cells can be engineered to secrete anti-PD-1 single-chain variable fragments (scFvs) locally, achieving high concentrations of checkpoint inhibitor within the tumor while minimizing systemic exposure. This “cell-as-drug-factory” approach reduces immune-related adverse events and has shown enhanced efficacy in preclinical solid tumor models (190) (**Figure 4**).

### 4.2 Clinical translation: emerging breakthroughs in overcoming immunosuppression

The core bottlenecks in clinical translation are efficacy heterogeneity, adaptive resistance, poor penetration into physically restrictive solid tumors and limited safety controllability. Emerging modalities—including precision biomarker stratification, local delivery, engineered cellular therapies, optimized treatment sequence and repurposed metabolic agents—should therefore be viewed as complementary strategies rather than universal solutions. Claims of clinical promise require separate consideration of preclinical activity, early pharmacodynamic evidence and demonstrated survival benefit in adequately designed trials.

#### 4.2.1 Biomarkers reflecting immunosuppressive cell burden for patient stratification

For clinical use, biomarker terminology must be explicit. Prognostic biomarkers associate with outcome independently of a particular intervention; predictive biomarkers identify differential likelihood of benefit from a defined therapy; pharmacodynamic biomarkers report on-target biological change during treatment; and exploratory biomarkers generate hypotheses that require prospective validation. The same suppressive-cell signal can occupy different categories across tumor types and treatment settings, so classification should be determined by study design rather than by assay technology alone.

The clinical efficacy of immunotherapy is fundamentally constrained by the immunosuppressive cell burden within the tumor microenvironment—comprising Treg, MDSCs, and TAMs--which can neutralize effector T-cell activity and drive treatment failure even in tumors with high PD-L1 expression or abundant CD8^+^ T-cell infiltration. Reliable biomarkers that quantify this immunosuppressive load are essential for accurate patient stratification, as conventional predictors such as PD-L1 immunohistochemistry and tumor mutational burden (TMB) capture only the immunostimulatory side of the immune equation and largely overlook the counterbalancing suppressive forces that dictate net antitumor immunity (2, 191). Circulating MDSC levels represent the most extensively validated liquid-biopsy biomarker in this domain. A meta-analysis encompassing 17 studies and 1,035 patients with solid tumors demonstrated that lower baseline circulating MDSC levels were associated with significantly improved overall survival (HR = 2.13; 95% CI 1.51–2.99) and progression-free survival (HR = 1.87; 95% CI 1.29–2.72) following ICI therapy, with the monocytic MDSC (M-MDSC) subpopulation emerging as a consistent prognostic marker across ethnicity, tumor type, and ICI target (192). In recurrent/metastatic head and neck squamous cell carcinoma, multivariate analysis revealed that circulating LOX-1^+^ PMN-MDSCs constitute an independent negative prognostic factor for pembrolizumab resistance--with high baseline levels predicting significantly worse survival--while CD137^+^ Tregs correlated with poor performance status and reduced therapeutic response (193). In breast cancer, elevated circulating CSF3R^+^ MDSCs have been identified as a predictive biomarker for early detection of tumor recurrence, with *in vitro* evidence confirming that these cells exhibit enhanced reactive oxygen species production and robust T-cell suppressive capacity (194).

Beyond individual cell populations, composite gene signatures derived from immunosuppressive cell transcriptomes are enabling multidimensional risk stratification. A single-cell RNA-sequencing analysis of breast cancer identified a 5-gene MDSC-associated risk score (BCL2A1, GDI2, GRINA, RNASE1, SERPINA1) that achieved robust prognostic stratification with a 1- to 10-year overall survival AUC exceeding 0.6 across TCGA-BRCA, METABRIC, and SCANB cohorts. High-risk patients exhibited an immunosuppressive tumor microenvironment enriched in M2 macrophages and Tregs with diminished cytotoxic T-lymphocyte activity, and showed poor responses to both chemotherapy and immunotherapy (195). In clear cell renal cell carcinoma, a prognostic signature derived from MDSC- and Treg-related differentially expressed genes effectively discriminated between immune and clinical features, enabling the prediction of immunotherapy outcomes (196). For Tregs specifically, a multi-omics pan-cancer analysis of effector versus resting Treg gene signatures—defined by FOXP3, CTLA-4, CCR8, and TNFRSF9 expression—demonstrated that effector Treg enrichment is associated with divergent survival outcomes across tumor types, underscoring the functional heterogeneity that must be accounted for when interpreting Treg infiltration as a biomarker. The FOXP3/CD8^+^ ratio has similarly been validated as an independent risk factor for postoperative recurrence in NSCLC, highlighting the importance of balancing effector and regulatory populations in prognostic assessment (197).

Spatial profiling technologies are redefining how immunosuppressive cell burden is quantified, revealing that the biological significance of these cells depends critically on their anatomical localization and functional context rather than absolute abundance alone. Spatial transcriptomic analysis of hepatocellular carcinoma demonstrated that hypoxic metabolic myofibroblastic cancer-associated fibroblasts (hmmyCAFs) accumulate at the invasive front, forming collagen-rich physical barriers that exclude CD8^+^ T cells while secreting POSTN to suppress immune checkpoint signaling—a spatial architecture that correlates with reduced progression-free survival and immunotherapy resistance (198). In EGFR-mutant versus KRAS-mutant NSCLC, spatial transcriptomics revealed a significantly reduced proportion of pro-inflammatory macrophages in the stromal compartment of EGFR-mutant tumors, coupled with lower immune gene set enrichment scores, identifying a spatially defined immunosuppressive signature that may explain the differential ICI sensitivity between these oncogene-driven subsets (199). Integrating multi-omics approaches, MARCKS expression was found to be enriched in myeloid cell populations within the HCC microenvironment and functionally associated with enhanced JAK/STAT3 signaling and M2-like macrophage polarization, positioning MARCKS as a spatially informed biomarker of TAM-mediated immunosuppression (200). The convergence of these spatial and molecular approaches with liquid biopsy—where ctDNA dynamics can be correlated with changes in circulating immunosuppressive cell levels to provide a comprehensive, minimally invasive window into the evolving tumor–immune interplay—is establishing a new paradigm in which immunosuppressive cell burden can be assessed longitudinally and non-invasively, enabling dynamic risk-adapted therapeutic decision-making that matches treatment intensity to the prevailing immunosuppressive landscape (201, 202) (**Table 1**).

**Table 1:** Biomarkers reflecting immunosuppressive cell burden for patient stratification.

Biomarker type	Sample source	Key findings	Clinical significance	Key references
Circulating MDSC levels (especially M-MDSC subset)	Peripheral blood	Lower baseline MDSCs associated with better OS (HR=2.13) and PFS (HR=1.87) (17 studies, 1,035 patients)	Liquid biopsy marker independently predicting ICI efficacy	(4)
LOX-1^+^ PMN-MDSCs	Peripheral blood	High baseline levels an independent negative prognostic factor for pembrolizumab resistance	Predicts immunotherapy resistance in head & neck squamous cell carcinoma, etc.	(5)
CSF3R^+^ MDSCs	Peripheral blood	Elevated levels predict early breast cancer recurrence; confirmed T-cell suppressive capacity *in vitro*	Recurrence monitoring and efficacy prediction	(6)
5-gene MDSC risk score (BCL2A1, GDI2, GRINA, RNASE1, SERPINA1)	Tumor tissue (scRNA-seq)	High risk score correlates with M2 macrophage/Treg enrichment and reduced CD8^+^ T cell activity; 1- to 10-year AUC >0.6 across multiple cohorts	Guides chemotherapy and immunotherapy decisions in breast cancer	(7)
MDSC/Treg-related differential gene expression signature	Tumor tissue	Effectively discriminates immune/clinical features of ccRCC; predicts immunotherapy outcomes	Prognostic stratification for renal cancer immunotherapy	(8)
Effector Treg gene signature (FOXP3, CTLA-4, CCR8, TNFRSF9)	Tumor tissue (multi-omics)	Effector Treg enrichment shows divergent survival associations across cancer types	Functional subset interpretation needed for Treg infiltration as biomarker	(9)
FOXP3/CD8^+^ ratio	Tumor tissue	Independent risk factor for postoperative recurrence in NSCLC	Balances effector/regulatory cells for prognostic assessment	(10)
Spatial transcriptomic signature: hmmyCAFs (POSTN^+^, collagen-rich)	Tumor tissue (spatial transcriptomics)	Forms physical barrier at invasive front of HCC, excludes CD8^+^ T cells, suppresses immune checkpoint signaling	Spatial tissue marker indicating immunotherapy resistance	(11)
MARCKS expression (enriched in myeloid cells)	Tumor tissue (integrated multi-omics)	Enhances JAK/STAT3 signaling and M2-like macrophage polarization	Spatially informed marker of TAM-mediated immunosuppression	(13)
Dynamic ctDNA combined with circulating immunosuppressive cells	Liquid biopsy	Longitudinal assessment of evolving tumor-immune interplay	Enables dynamic risk-adapted therapeutic decision-making	(14, 15)

The following table provides a practical framework for interpreting biomarkers of immunosuppressive cell burden and separating mature clinical applications from exploratory spatial and dynamic monitoring approaches (**Table 2**).

**Table 2:** Biomarker categories for immunosuppressive cell burden.

Biomarker class	Suppressive population/axis	Detection method	Interpretive role	Representative context	Evidence level
Circulating cell burden	MDSCs; activated Tregs	Flow cytometry/blood immunophenotyping	Predictive or pharmacodynamic candidate	ICI-treated solid tumours	Most mature; assay and threshold harmonization still needed
Tissue immune composition	TAM, Treg and MDSC abundance	IHC, multiplex IF or bulk RNA signatures	Prognostic and hypothesis-generating predictive marker	Tumor-specific	Context-dependent; requires clinical validation
Spatial suppressive architecture	Myeloid–CAF–T-cell exclusion niches	Spatial transcriptomics/multiplex imaging	Exploratory/patient-selection candidate	Stroma-rich or immune-excluded tumours	Emerging; requires standardized spatial metrics
Dynamic composite monitoring	Suppressive-cell burden + ctDNA/imaging	Serial liquid biopsy and imaging	Pharmacodynamic or adaptive-treatment candidate	Longitudinal treatment setting	Emerging; prospective utility unproven

The table distinguishes prognostic, predictive, pharmacodynamic and exploratory uses; evidence level reflects the degree of clinical validation rather than biological plausibility alone.

#### 4.2.2 Novel agents and engineered cell therapies that directly target or reprogram immunosuppressive cells

The therapeutic targeting of immunosuppressive cells within the tumor microenvironment—rather than tumor cells themselves—represents a paradigm shift in overcoming immunotherapy resistance. Unlike conventional immunotherapies that seek to amplify effector T-cell responses, this class of agents aims to dismantle the immune-suppressive infrastructure established by Tregs, MDSCs, and TAMs, thereby restoring the host’s intrinsic antitumor immunity. Current strategies span selective depletion of suppressive populations, functional blockade of their effector mechanisms, and--most notably--reprogramming of these cells toward immunostimulatory phenotypes, with several agents now advancing through early-to late-phase clinical trials (203, 204).

##### 4.2.2.1 Targeting Tregs: selective depletion and functional reprogramming

The selective elimination of intratumoral Tregs while preserving peripheral immune homeostasis has been a long-standing goal in cancer immunotherapy. Anti-CCR8 antibodies have emerged as a clinically tractable strategy, leveraging the preferential expression of CCR8 on tumor-infiltrating Tregs. CHS-114, a highly selective cytolytic anti-CCR8 monoclonal antibody, demonstrated robust and selective depletion of CCR8^+^ Tregs with favorable immune remodeling--including increased CD8^+^ T-cell infiltration--in participants with advanced solid tumors in an ongoing Phase 1b trial as both monotherapy and in combination with toripalimab, with the clinical program expanding to include colorectal cancer (205). DT-7012, a differentiated Treg-depleting anti-CCR8 antibody, entered Phase I/II DOMISOL clinical study in October 2025, with subsequent expansion of enrollment to European sites, marking significant global development progress for this class. Beyond depletion-based approaches, Treg reprogramming represents an emerging frontier. A bispecific antibody-drug conjugate targeting CCR8 and TNFR2--two surface markers predominantly co-expressed on tumor-infiltrating Tregs--elicited potent antitumor efficacy through selective delivery of cytotoxic payloads to dual-positive Tregs in preclinical models (206). In a complementary approach, the immunocytokine αTIGIT-IL2, generated by fusing IL-2 to an anti-TIGIT monoclonal antibody, selectively targeted TIGIT^+^ Tregs and promoted their functional fragility within the tumor milieu, resulting in robust antitumor immunity and tumor regression in both immune checkpoint blockade-sensitive and -resistant mouse models, including triple-negative breast cancer (207). This strategy of converting Tregs from a suppressive to a pro-inflammatory state represents a conceptually distinct alternative to Treg depletion.

##### 4.2.2.2 Targeting MDSCs: depletion, differentiation, and functional inhibition

MDSCs contribute to immune evasion through multiple parallel mechanisms--arginase-1-mediated arginine depletion, reactive oxygen species production, and expansion of Treg populations--making them attractive yet challenging therapeutic targets (208). Current strategies against MDSCs encompass four modalities: depletion, functional inhibition, blockade of tumor recruitment, and induction of differentiation into mature, non-suppressive myeloid cell. Among the most clinically advanced are agents that redirect MDSC differentiation. All-trans retinoic acid (ATRA) has been shown to promote the differentiation of MDSCs into mature dendritic cells and macrophages, reducing their suppressive capacity. A phase I/II clinical trial has combined ATRA with pembrolizumab in advanced melanoma, and it serves as a key proof-of-concept for differentiation therapy against MDSCs. Disruption of MDSC recruitment via chemokine receptor blockade represents another clinically viable strategy, with CCR2 and CCR5 inhibitors under investigation to prevent monocytic MDSC trafficking to tumors (209). Preclinically, the identification of MARCO as a critical regulator of MDSC differentiation and immunosuppression, along with the ApoE/LDLR axis as a mediator of MDSC-driven systemic immunosuppression, has opened additional therapeutic avenues currently progressing toward clinical evaluation (210, 211).

##### 4.2.2.3 Targeting TAMs: reprogramming, phagocytic checkpoint blockade, and CAR-based platforms

TAMs represent the most abundant immunosuppressive population in many solid tumors, and therapeutic strategies have evolved from broad depletion to more nuanced reprogramming approaches that preserve or enhance their antitumor potential. CSF1R inhibition selectively targets tumor-promoting macrophages while sparing antitumor populations, with sustained CSF1R inhibition shown to augment antitumor immunity through TAM reprogramming--reducing CD206 expression and IL-10 secretion while enhancing phagocytic activity (212, 213). The macrophage checkpoint CD47-SIRPα axis, which delivers a “don’t eat me” signal that enables tumor cells to evade phagocytosis, has been targeted by agents such as magrolimab and lemzoparlimab, with multiple clinical trials evaluating these agents in combination with existing immunotherapies across solid tumors and hematologic malignancies (214, 215).

A transformative advance in this domain is the development of CAR-based platforms that directly target immunosuppressive myeloid cells. TREM2, a myeloid checkpoint selectively expressed on immunosuppressive TAMs, has been leveraged for both CAR-macrophage and CAR-monocyte therapies. In preclinical studies, TREM2-directed CAR-monocytes demonstrated TREM2-specific phagocytic activity, significantly reduced breast tumor growth, and enhanced CD8^+^ T-cell infiltration and immune checkpoint blockade sensitivity in orthotopic triple-negative breast cancer models (216). A parallel approach engineering TREM2-targeted CAR-T cells to eliminate TAMs showed robust *in vivo* antitumor efficacy and is advancing toward clinical evaluation (217). In the clinical setting, CT-0508, an autologous anti-HER2 CAR-M therapy, demonstrated feasibility, safety, and preliminary signs of TME remodeling in patients with HER2-overexpressing advanced solid tumors in a first-in-human Phase 1 trial, including evidence of TAM reprogramming toward a pro-inflammatory phenotype (218). An ongoing clinical trial of CAR-M in metastatic HER2-low and HER2-positive solid tumors has enrolled patients for intraperitoneal administration, further establishing the clinical viability of myeloid cell-based cellular immunotherapy.

##### 4.2.2.4 Emerging modalities: immunostimulatory ADCs, engineered cytokines, and tumor microenvironment remodeling

The convergence of antibody-drug conjugate technology with immunotherapy has yielded agents that simultaneously deliver cytotoxic payloads and immunostimulatory signals within the TME. DB-1419, a bifunctional bispecific anti-B7-H3/PD-L1 ADC, demonstrated the capacity to simultaneously target tumor cells and immunosuppressive checkpoint pathways in preclinical studies, while DS3610, a first-in-class STING agonist ADC developed by Daiichi Sankyo, entered clinical development to activate innate immune sensing within the TME—a strategy that may reprogram TAMs and overcome myeloid-mediated immunosuppression (219). Engineered cytokines with Treg-sparing properties, such as MDNA11, a long-acting IL-2 super-agonist that selectively activates IL-2Rβ without engaging CD25, preferentially expand cytotoxic T cells and NK cells while avoiding Treg expansion, and are currently under evaluation in the Phase I/II ABILITY-1 trial (220).

#### 4.2.3 Combination regimens designed to overcome immunosuppressive barriers

The clinical reality that single-agent immunotherapy yields durable responses in only a minority of patients is fundamentally attributable to the redundant and complementary immunosuppressive networks operating within the tumor microenvironment. Tregs, MDSCs, and TAMs do not act in isolation—they establish mutually reinforcing circuits through shared metabolic pathways, cytokine crosstalk, and coordinated exclusion of effector T cells. Consequently, effective combination strategies must be designed not simply to amplify T-cell activation, but to systematically dismantle the specific immunosuppressive barriers operative in a given tumor. This principle has driven a paradigm shift from empirical “drug stacking” to mechanism-driven “synergistic targeting of immunosuppressive axes,” with combinations now rationally assembled to concurrently deplete suppressive populations, reprogram their functional phenotype, and restore effector cell access to tumor parenchyma (221).

##### 4.2.3.1 Targeting TAM-mediated immune exclusion and phagocytic evasion

TAMs constitute the most abundant immunosuppressive population in many solid tumors and contribute to immune evasion through two principal mechanisms: exclusion of CD8^+^ T cells from tumor nests and engagement of the CD47-SIRPα “don’t eat me” checkpoint to evade phagocytosis. CSF1R inhibition combined with PD-1/PD-L1 blockade represents the most clinically advanced strategy to disrupt this axis. A Phase I trial combining the CSF1R inhibitor vimseltinib with avelumab in advanced sarcomas--tumors characterized by dense macrophage infiltration and near-universal ICI resistance--established a recommended Phase II dose and demonstrated pharmacodynamic evidence of target engagement, with peripheral blood analysis revealing reductions in both MDSC and Treg populations alongside RNA-sequencing evidence of increased tumor-infiltrating T cells. Although objective responses were not observed (median PFS 1.55 months), a subset of leiomyosarcoma patients achieved a median overall survival of 18.19 months, and patients with higher baseline CD3^+^ T-cell counts or elevated CD8^+^/Treg ratios derived greater clinical benefit, providing biomarker leads for patient enrichment (222). In metastatic triple-negative breast cancer, the combination of CSF1R inhibition with the chemotherapy agent eribulin was shown in a translational study to relieve systemic immune suppression and expand the therapeutic index of PD-1 blockade--approximately 60% of mice bearing transgenic mammary tumors experienced sustained tumor control--establishing a clinical rationale for chemo-immunotherapy-CSF1R inhibitor triplets (223). In preclinical pancreatic cancer models, a TAM-targeted nanotherapy platform delivering either a CSF1R inhibitor or the STING agonist cGAMP demonstrated that while CSF1R inhibitor-loaded nanoparticles efficiently depleted TAMs and produced moderate antitumor effects, cGAMP-loaded nanoparticles reprogrammed TAMs into immune-stimulating effectors with superior tumor control, suggesting that functional reprogramming rather than wholesale depletion may be the more effective strategy (224).

The macrophage checkpoint CD47-SIRPα axis has been targeted through multiple modalities. Selective SIRPα blockade demonstrated higher response rates in solid tumors compared to anti-CD47 monoclonal antibody combinations in meta-analyses, with a more favorable grade 1–2 toxicity profile, particularly regarding hematologic adverse events that have historically limited the development of this class (225). A mesothelin-directed SIRPα-αMSLN bifunctional antibody construct achieved tumor-restricted CD47-SIRPα blockade, inducing NK cell-mediated cytotoxicity and macrophage phagocytosis while preventing on-target off-tumor toxicities—a precision approach that may broaden the therapeutic window of phagocytic checkpoint blockade in solid tumors (226).

##### 4.2.3.2 Eliminating MDSCs through multimodal targeting

Given that MDSCs employ parallel suppressive mechanisms-arginine depletion via arginase-1, reactive oxygen species production, and Treg expansion-effective elimination requires concurrent targeting of their recruitment, survival, and effector function. Therapeutic strategies under clinical investigation encompass four modalities: depletion, functional inhibition, blockade of tumor trafficking, and induction of differentiation into mature, non-suppressive myeloid cells. ATRA, which promotes MDSC differentiation into mature dendritic cells and macrophages, has been evaluated in combination with pembrolizumab in a Phase I/II trial in advanced melanoma as a proof-of-concept for differentiation therapy (227). Disruption of MDSC recruitment via CCR2 and CCR5 inhibition represents a complementary clinical strategy to prevent monocytic MDSC trafficking to tumors (227). A critical mechanistic insight emerging from these studies is that MDSC-targeted monotherapy is unlikely to produce clinical responses-the redundancy of their suppressive programs necessitates concurrent PD-1/PD-L1 blockade to enable effector T-cell function once the myeloid brake is released.

##### 4.2.3.3 Treg depletion as a combination partner

The selective elimination of intratumoral Tregs via CCR8 targeting has advanced rapidly into clinical evaluation in combination with PD-1 inhibitors. Cafelkibart (LM-108), an Fc-optimized anti-CCR8 monoclonal antibody designed to selectively deplete tumor-infiltrating Tregs while sparing peripheral Tregs, was evaluated in combination with anti-PD-1 therapy in 80 patients with advanced pancreatic cancer who had progressed on at least one prior line of therapy--including 17.5% with prior anti-PD-1 exposure and 65.0% with baseline liver metastases. The combination achieved an objective response rate of 20.3% (95% CI 11.8–31.2%), a disease control rate of 62.2%, and a median overall survival of 10.02 months, with grade ≥3 treatment-related adverse events in 52.5% of patients, the most common being lipase elevation (7.5%), ALT elevation (6.3%), and AST elevation (5.0%). Among patients who had received only one prior line of therapy, the ORR reached 24.4%, suggesting greater benefit when deployed earlier in the treatment course. In parallel, CHS-114, a highly selective cytolytic anti-CCR8 antibody, demonstrated a confirmed partial response in a heavily pretreated PD-1-refractory patient, accompanied by >50% depletion of CCR8^+^ Tregs and a corresponding increase in CD8^+^ T cells—providing clinical proof of mechanism—with the program subsequently expanding to include head and neck squamous cell carcinoma and colorectal cancer cohort.

##### 4.2.3.4 Radiotherapy combined with immunotherapy: exploiting and neutralizing myeloid responses

The temporal relationship between radiotherapy and immunotherapy has emerged as a critical determinant of therapeutic efficacy, with the mechanistic basis for this timing effect now being elucidated at the level of immunosuppressive myeloid cell dynamics. Radiotherapy, while capable of inducing immunogenic cell death and releasing tumor antigens, paradoxically activates and recruits immunosuppressive myeloid cells including MDSCs and TAMs, thereby creating a window of immune vulnerability. The immune checkpoint VISTA has been identified as a central mediator of this radiotherapy-induced immunosuppression: VISTA is highly expressed on myeloid cells in the tumor microenvironment of both murine and human head and neck cancers, and radiotherapy further increases the abundance of VISTA^+^ myeloid cells in both the tumor and circulation (228). Genetic deletion or antibody-mediated blockade of VISTA--combined with either fractionated or ablative radiotherapy—significantly enhanced tumor control compared to either modality alone across multiple preclinical models (head and neck cancer, breast cancer, and colorectal cancer), with scRNA-seq analysis revealing that VISTA blockade fundamentally reprogrammed macrophages and neutrophils from a tumor-supportive to an antitumorigenic state, resulting in augmented intratumoral T-cell function (228). These findings provide a mechanistic framework for the emerging clinical principle of sequential rather than concurrent immunotherapy-radiotherapy combinations: delivering immunotherapy after radiotherapy permits exploitation of antigen release while avoiding concomitant suppression of effector T cells by radiotherapy-recruited myeloid populations. This paradigm is further supported by the negative results of multiple randomized trials of concurrent chemoradiotherapy with immunotherapy in lung cancer, head and neck cancer, and cervical cancer, where the addition of ICIs to radiotherapy consistently failed to improve outcomes--likely due to myeloid-mediated suppression of the very T cells the ICIs sought to activate (229).

##### 4.2.3.5 Dual and multi-specific antibody platforms

The convergence of multi-specific antibody engineering with immunosuppressive cell targeting is yielding single-agent combination therapies capable of simultaneously engaging innate and adaptive immunity while neutralizing immunosuppressive cytokines. HCB301, a first-in-class trispecific fusion protein simultaneously targeting SIRPα-CD47, PD-1/PD-L1, and TGF-β, demonstrated robust immune activation with enhanced macrophage-mediated phagocytosis, restoration of T-cell effector function, and superior tumor growth inhibition compared to monofunctional or bifunctional controls in preclinical head and neck squamous cell carcinoma and triple-negative breast cancer models. By integrating PD-1 blockade and TGF-β neutralization into a single molecule with CD47-SIRPα disruption, HCB301 exemplifies a modular approach to overcoming the three dominant immunosuppressive barriers--phagocytic evasion, T-cell exhaustion, and cytokine-mediated suppression—through a single therapeutic entity. The LAG-3/TIGIT bispecific antibody ZGGS15 has entered first-in-human studies, demonstrating capacity to reverse Treg-mediated inhibition of both T cells and NK cell. In the neoadjuvant setting for resectable NSCLC, an umbrella trial evaluating tislelizumab with anti-TIGIT (ociperlimab) or anti-LAG-3 (LBL-007) plus chemotherapy demonstrated that in PD-L1 TC ≥50% patients, the anti-TIGIT combination achieved a major pathological response rate of 100% in the non-squamous NSCLC subgroup (6/6 patients), highlighting the potential of next-generation checkpoint combinations in specific molecular and histological contexts (230).

##### 4.2.3.6 Epigenetic priming to overcome immunosuppressive barriers

Epigenetic modulation is emerging as a strategy to dismantle immunosuppressive barriers at the transcriptional level, with histone deacetylase inhibitors (HDACi) demonstrating capacity to simultaneously down-regulate Tregs while up-regulating tumor antigen presentation machinery. In triple-negative breast cancer, HDAC inhibition was shown to down-regulate CD4^+^Foxp3^+^ Tregs *in vitro* and, when combined with PD-1/CTLA-4 blockade in the 4T1 syngeneic mouse model, resulted in significantly decreased tumor growth and increased survival, associated with enhanced T-cell tumor infiltration and a reduction in intratumoral Tregs (231). Novel HDAC inhibitors such as GCJ-490A have demonstrated synergy with anti-PD-1 antibodies in both breast and lung cancer syngeneic models through TME remodeling (232), while the class I HDAC inhibitor chidamide enhanced chromatin accessibility at immune-related gene promoters, augmenting the antitumor efficacy of immunotherapy in solid tumors through vascular normalization (233).

#### 4.2.4 Repurposed drugs and pan-cancer strategies for immunosuppressive cell modulation

The oncology paradigm has increasingly shifted from histology-driven approaches toward biomarker-defined precision strategies, with tumor-agnostic approvals marking a decisive step in this transition. Since the initial FDA approval of pembrolizumab for MSI-H/dMMR solid tumors in 2017, the list of tissue-agnostic therapies has expanded to encompass NTRK inhibitors (larotrectinib and entrectinib), dostarlimab (dMMR solid tumors), pembrolizumab for TMB-H solid tumors, the dabrafenib plus trametinib combination for BRAF V600E-mutant tumors, selpercatinib for RET fusion-positive tumors, and the HER2-targeted ADC fam-trastuzumab deruxtecan (233). Notably, the latest tumor-agnostic approval for HER2 IHC 3^+^ solid tumors marks the expansion of pan-cancer immunotherapy from immune checkpoint inhibition into the domain of ADCs, demonstrating that the molecular stratification principle is now being systematically applied across multiple therapeutic modalities (234). These developments have in turn catalyzed the evolution of clinical trial architectures from single-arm biomarker-driven basket studies to adaptive multi-arm umbrella designs that enable simultaneous evaluation of multiple targeted agents across histologically diverse patient populations sharing defined molecular alterations (235).

Drug repurposing has emerged as a complementary strategy to expand the reach of pan-cancer immunotherapy while circumventing the protracted timelines and high costs associated with *de novo* drug development. The core rationale lies in modulating the immunosuppressive tumor microenvironment--reprogramming tumor-associated macrophages from an M2 to an M1 phenotype, inhibiting the infiltration of regulatory T cells and myeloid-derived suppressor cells, and reducing immunosuppressive metabolites—thereby converting immunologically “cold” tumors into “hot” tumors that are more susceptible to immune checkpoint blockade (236). Among the most extensively investigated candidates, the COX-2/PGE2 axis has been established as an independent immune checkpoint: celecoxib, by inhibiting COX-2, reduces PGE2 synthesis, attenuates its chemotactic effects on Tregs and MDSCs, and promotes effector T cell infiltration into tumor tissue (237). Recent studies have further shown that PGE2 suppresses anticancer immunity through downregulation of IL-2 receptors and consequent inhibition of mitochondrial metabolism in T cells, providing a refined mechanistic basis for COX-2 inhibition as an adjunct to immunotherapy (238). Retrospective studies have suggested improved survival outcomes in non-small cell lung cancer and colorectal cancer patients receiving selective COX-2 inhibitors in combination with immune checkpoint inhibitors, and prospective validation trials are ongoing to establish causality (239).

Metformin exerts its immunomodulatory effects predominantly through the AMPK pathway, enhancing the antitumor immune functions of CD8^+^ T cells and γδ T cells (240). Systematic reviews have elucidated that metformin remodels CD8^+^ T cell metabolic programming via AMPK/mTOR pathways--enhancing oxidative phosphorylation and fatty acid oxidation—thereby improving T cell exhaustion, promoting memory formation, and augmenting the efficacy of immune checkpoint inhibitors and adoptive cell therapies across diverse pathological contexts including tumors, chronic infections, and inflammatory diseases (241). A recent study demonstrated that metformin improves lung cancer outcomes and enhances immunotherapy efficacy in an obesity-specific manner, suggesting a biomarker-informed direction for its precision application (242). Conversely, β-adrenergic blockade has shown promise in preclinical models, but a multicenter retrospective study and subsequent meta-analysis involving over 4,000 patients with solid tumors found no statistically significant survival benefit when beta-blockers were added to immune checkpoint inhibitors, underscoring the critical importance of prospective validation and the potential gap between mechanistic plausibility and clinical translation (243).

The convergence of pan-cancer expansion and drug repurposing is giving rise to entirely new combination strategies--integrating tissue-agnostic immunotherapy with the immunomodulatory effects of concomitant medications. AI-based platforms are accelerating this translational process by integrating multi-omics data to predict drug-target interactions and optimize individualized combination regimens, enabling repurposed drugs to be embedded into the pan-cancer immunotherapy framework as “universal immune enhancers” (244). Representative examples include the phase II trial of rintatolimod in combination with durvalumab for advanced pancreatic cancer and the phase III trial of IFx-2.0 in combination with pembrolizumab for Merkel cell carcinom. The validation of causality through prospective randomized controlled trials, AI-assisted optimization of individualized combination regimens, and precise management of the benefit-toxicity balance represent critical nodes that urgently require breakthroughs in this intersecting field (**Table 3**).

**Table 3:** Representative repurposed drugs with immunomodulatory mechanisms and clinical evidence.

Drug/Strategy	Original indication	Immunomodulatory mechanism	Effect on immunosuppressive cells	Clinical evidence status	Key references
Celecoxib	Arthritis, pain	COX-2 inhibition → reduces PGE_2_ synthesis	Attenuates PGE_2_ chemotaxis of Tregs and MDSCs; promotes effector T cell infiltration	Retrospective studies suggest improved survival in NSCLC and CRC; prospective validation ongoing	(5, 6)
Metformin	Type 2 diabetes	Activates AMPK pathway; reprograms CD8^+^ T cell metabolism	Enhances antitumor function of CD8^+^ T and γδ T cells; ameliorates T cell exhaustion and promotes memory formation	Preclinical and retrospective support; obesity-specific improved outcomes in lung cancer	(7, 8)
β-blockers	Hypertension, arrhythmia	Inhibits β-adrenergic signaling	Preclinical models show reduced immunosuppression; however, no survival benefit in clinical studies	Multicenter retrospective and meta-analysis (>4,000 patients) showed no statistical benefit with ICI combination	(9, 10)
Rintatolimod	Chronic fatigue syndrome (immunomodulator)	TLR3 agonist	Not directly defined; enters Phase II trial in pancreatic cancer combined with durvalumab	Phase II ongoing	(13)
IFx-2.0	Immunotherapy (bacterial vaccine)	Expresses emm55 antigen; activates T cells	Combined with pembrolizumab for Merkel cell carcinoma	Phase III ongoing	(14)
Pan-cancer (tumor-agnostic) strategies					
Pembrolizumab (MSI-H/dMMR, TMB-H)	Multiple solid tumors	Blocks PD-1	Releases T cell inhibition; indirectly reduces Treg/TAM immunosuppression	FDA-approved (2017, 2020)	(1, 2)
Trastuzumab deruxtecan (HER2 IHC 3+)	Breast, gastric cancer, etc.	ADC (HER2-targeted + topoisomerase inhibitor)	Induces immunogenic cell death; remodels TME	First tumor-agnostic ADC approval (2024)	(2)
Dabrafenib + Trametinib (BRAF V600E)	Melanoma, etc.	MAPK pathway inhibition	Downregulates immunosuppressive cytokines; increases T cell infiltration	Tumor-agnostic approval	(1)
Selpercatinib (RET fusion)	Lung, thyroid cancer, etc.	RET inhibitor	Not fully elucidated; approved for agnostic use	Tumor-agnostic approval	(1)
Celecoxib	Arthritis, pain	COX-2 inhibition → reduces PGE_2_ synthesis	Attenuates PGE_2_ chemotaxis of Tregs and MDSCs; promotes effector T cell infiltration	Retrospective studies suggest improved survival in NSCLC and CRC; prospective validation ongoing	(5, 6)

## 5 Conclusions and future perspectives

This review synthesizes immunosuppressive cells in the tumor microenvironment as a dynamic, tissue-conditioned network rather than a catalog of individual populations. MDSCs, Tregs, TAMs and additional suppressive subsets converge on recurring resistance modules—metabolic competition, inhibitory signaling, impaired antigen presentation, stromal exclusion and therapy-induced remodeling—while the dominant module varies across tumor sites and between primary and metastatic lesions. This perspective supports mechanism-matched combinations but also emphasizes that much of the field remains preclinical or context-dependent. Clinical progress will require careful evidence grading, biomarker-defined patient selection, measurement of target engagement, feasible access to the relevant tumor compartment and prospective demonstration that added biological activity yields a meaningful benefit-toxicity balance.

Looking forward, the field is poised to move in broader and more transformative directions. First, from targeting individual cells to modulating the ecosystem. The immunosuppressive microenvironment should be viewed not as a collection of isolated targets but as a dynamic ecosystem. Future strategies will need to simultaneously address reciprocal interactions among immune cells, stroma, metabolites, neural signals, and microbial communities, thereby reshaping the global “climate” of the microenvironment rather than merely eliminating one suppressive population (245). Second, from static biomarkers to dynamic adaptive therapy. Current baseline biomarkers (e.g., PD-L1, TMB) cannot capture real-time changes in immunosuppressive burden during treatment. The future lies in integrating liquid biopsy (circulating suppressive cells, ctDNA), radiomics, and real-time immune monitoring to establish an adaptive framework in which immunotherapies are adjusted on demand according to the evolving state of the tumor microenvironment – a truly personalized, dynamic approach (246). Third, from tissue-centric to pan-cancer universal principles. Different tumor types share conserved immunosuppressive logic (e.g., specific metabolic axes or chemokine networks), whereas the same histology can display divergent suppressive patterns across patients. Deciphering both the “common modules” and “context-specific variables” of immunosuppression across cancer types will enable the development of strategies that are broadly applicable yet precisely stratifiable. Fourth, from treating the tumor to restoring immune homeostasis. The ultimate goal of curative immunotherapy is not only tumor elimination but also the restoration of normal immune surveillance and tolerance. This longer-term vision will require greater attention to higher-order regulatory axes such as immune senescence, chronic inflammation, and neuro-immune crosstalk (247), embedding anti-cancer immunotherapy within the broader framework of holistic health management (248). Achieving these goals will demand deep integration of multi-omics technologies, artificial intelligence, dynamic modeling, and innovative clinical trial designs – marking a paradigm shift from precision strikes to systemic repair in cancer immunotherapy (249).

## Glossary

ACT: Adoptive cell therapy
ADC: Antibody-drug conjugate
AhR: Aryl hydrocarbon receptor
AKT: Protein kinase B
ALDH1A1: Aldehyde dehydrogenase 1A1
AMPK: AMP-activated protein kinase
ARG1: Arginase 1
ASL: Argininosuccinate lyase
ATRA: All-trans retinoic acid
Breg: Regulatory B cell
CAF: Cancer-associated fibroblast
CAR: Chimeric antigen receptor
CAR-M: CAR-macrophage
CAR-NKT: CAR-natural killer T cell
CAR-T: CAR-T cell
CCL: Chemokine (C-C motif) ligand
CCR: C-C chemokine receptor
cGAS: Cyclic GMP-AMP synthase
CNS: Central nervous system
COX-2: Cyclooxygenase-2
CRT: Calreticulin
CSF1R: Colony-stimulating factor 1 receptor
CTLA-4: Cytotoxic T-lymphocyte-associated protein 4
CXCL: C-X-C motif chemokine ligand
CXCR: C-X-C chemokine receptor
DAMP: Damage-associated molecular pattern
DC: Dendritic cell
dMMR: Deficient mismatch repair
DN1: Double-negative 1 (B cell subset)
DP: Double-positive (T cell)
EMT: Epithelial-mesenchymal transition
EZH2: Enhancer of zeste homolog 2
FAO: Fatty acid oxidation
FATP2: Fatty acid transport protein 2
Foxp3: Forkhead box P3
GARP: Glycoprotein A repetitions predominant
GPR: G protein-coupled receptor
GVHD: Graft-versus-host disease
HCC: Hepatocellular carcinoma
HDAC: Histone deacetylase
HER2: Human epidermal growth factor receptor 2
HIF-1α: Hypoxia-inducible factor 1-alpha
HMGB1: High-mobility group box 1
ICI: Immune checkpoint inhibitor
ICD: Immunogenic cell death
ICOSL: Inducible T-cell costimulator ligand
IDO1: Indoleamine 2,3-dioxygenase 1
IFITM3: Interferon-induced transmembrane protein 3
ILC: Innate lymphoid cell
ILC2: Type 2 innate lymphoid cell
ILCreg: Regulatory innate lymphoid cell
IL-10: Interleukin-10
iNOS: Inducible nitric oxide synthase
JAK: Janus kinase
KLRG1: Killer cell lectin-like receptor G1
LAG-3: Lymphocyte-activation gene 3
LDH: Lactate dehydrogenase
LDLR: Low-density lipoprotein receptor
LOX-1: Lectin-type oxidized LDL receptor 1
LPS: Lipopolysaccharide
MAIT: Mucosal-associated invariant T cell
MARCKS: Myristoylated alanine-rich C-kinase substrate
MARCO: Macrophage receptor with collagenous structure
MDSC: Myeloid-derived suppressor cell
MerTK: MER tyrosine kinase
MHC: Major histocompatibility complex
MMP: Matrix metalloproteinase
MSI-H: Microsatellite instability-high
NET: Neutrophil extracellular trap
NK: Natural killer
NSCLC: Non-small cell lung cancer
PD-1: Programmed cell death protein 1
PDAC: Pancreatic ductal adenocarcinoma
PD-L1: Programmed death-ligand 1
PGE2: Prostaglandin E2
PPAR: Peroxisome proliferator-activated receptor
PRR: Pattern recognition receptor
ROS: Reactive oxygen species
SCFA: Short-chain fatty acid
scRNA-seq: Single-cell RNA sequencing
SIRPα: Signal regulatory protein alpha
SMS: Spermine synthase
SOCS: Suppressor of cytokine signaling
STAT: Signal transducer and activator of transcription
STING: Stimulator of interferon genes
TAA: Tumor-associated antigen
TAB: Tumor-associated inhibitory B cell
TAM: Tumor-associated macrophage
TAN: Tumor-associated neutrophil
TCF1: T-cell factor 1
TCR: T-cell receptor
TGF-β: Transforming growth factor-beta
TIGIT: T-cell immunoreceptor with Ig and ITIM domains
TIL: Tumor-infiltrating lymphocyte
TIM-3: T-cell immunoglobulin and mucin-domain containing-3
TLR: Toll-like receptor
TMB: Tumor mutational burden
TME: Tumor microenvironment
tolDC: Tolerogenic dendritic cell
Treg: Regulatory T cell
TREM2: Triggering receptor expressed on myeloid cells 2
TSLP: Thymic stromal lymphopoietin
VEGF: Vascular endothelial growth factor
VEGFR: Vascular endothelial growth factor receptor
VSIG4: V-set and immunoglobulin domain-containing 4.
